# Mix and match recognition modules for the formation of H-bonded duplexes†
†Electronic supplementary information (ESI) available: Detailed experimental procedures with spectroscopic characterization data, NMR titration protocols, titration spectra and results of fitting the data to a 1 : 1 binding isotherm. See DOI: 10.1039/c6sc01884j


**DOI:** 10.1039/c6sc01884j

**Published:** 2016-06-07

**Authors:** Alexander E. Stross, Giulia Iadevaia, Christopher A. Hunter

**Affiliations:** a Department of Chemistry , University of Cambridge , Lensfield Road , Cambridge CB2 1EW , UK . Email: herchelsmith.orgchem@ch.cam.ac.uk

## Abstract

Oligomeric molecules equipped with complementary H-bond recognition sites form stable duplexes in non-polar solvents. The use of a single H-bond between a good H-bond donor and a good H-bond acceptor as the recognition motif appended to a non-polar backbone leads to an architecture with interchangeable recognition alphabets. The interactions of three different families of H-bond acceptor oligomers (pyridine, pyridine N-oxide or phosphine oxide recognition module) with a family of H-bond donor oligomers (phenol recognition module) are compared. All three donor–acceptor combinations form stable duplexes, where the stability of the 1 : 1 complex increases with increasing numbers of recognition modules. The effective molarity for formation of intramolecular H-bonds that lead to zipping up of the duplex (EM) increases with decreasing flexibility of the recognition modules: 14 mM for the phosphine oxides which are connected to the backbone *via* a flexible linker; 40 mM for the pyridine N-oxides which have three fewer degrees of torsional freedom, and 80 mM for the pyridines where the geometry of the H-bond is more directional. However, the pyridine–phenol H-bond is an order of magnitude weaker than the other two types of H-bond, so overall the pyridine N-oxides form the most stable duplexes with the highest degree of cooperativity. The results show that it is possible to use different recognition motifs with the same duplex architecture, and this makes it possible to tune overall stabilities of the complexes by varying the components.

## Abstract 2

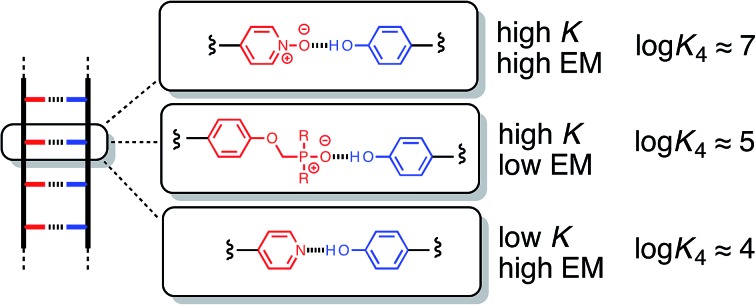
Equipping oligomeric molecules with different H-bond acceptor modules modulates the stabilities of the H-bonded duplexes formed in non-polar solvents.

## Introduction

**Fig. 1:**
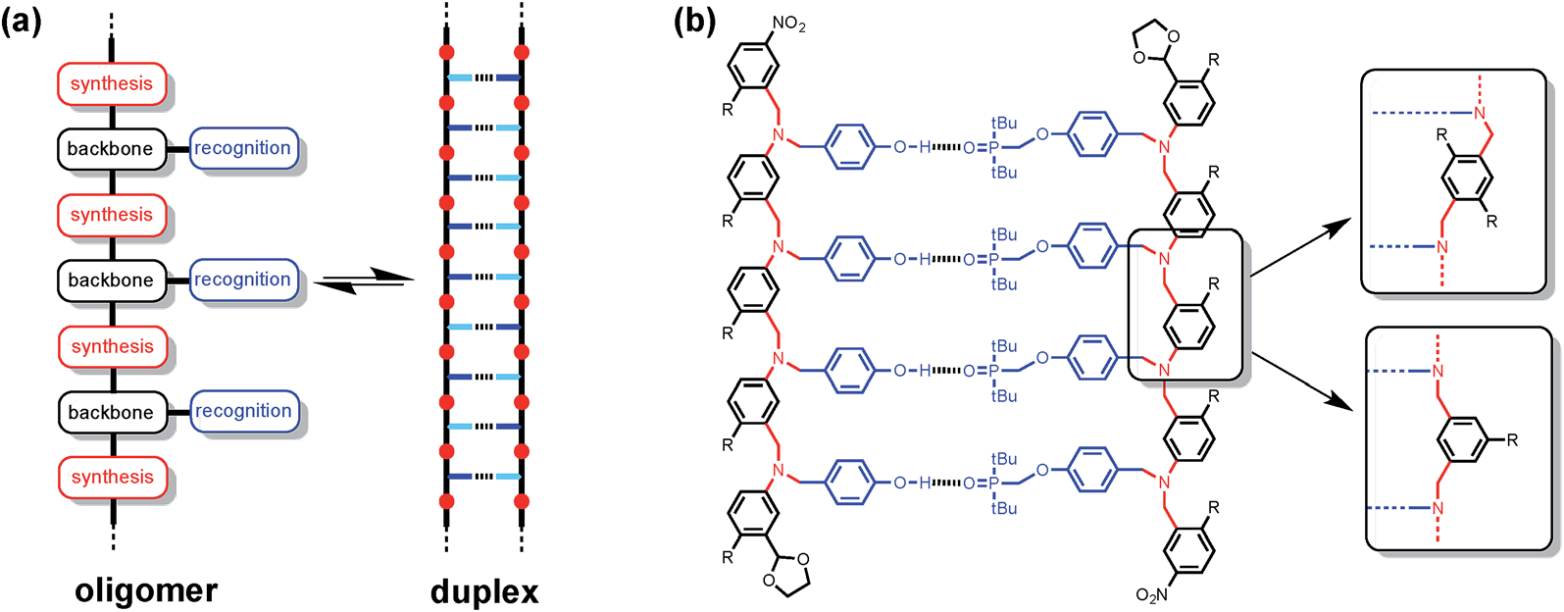
(a) Blueprint for a duplex-forming molecule. The key design components are the covalent chemistry used for synthesis (red), the non-covalent chemistry used for recognition (blue), the backbone linker that determines the geometric complementarity of the two chains (black). (b) The duplex formed by a phenol 4-mer (DDDD) and a phosphine oxide 4-mer (AAAA). Three possible backbone modules are shown. R is a 2-ethylhexoxy group that provides solubility in toluene. The antiparallel duplex is shown, but the parallel arrangement is also possible.

Synthetic oligomeric molecules equipped with a sequence of complementary recognition sites are capable of forming duplex structures reminiscent of the DNA double helix. Many different examples have been reported using metal ligand coordination, salt bridges, aromatic stacking interactions and H-bonding as the recognition sites.^1^ In most cases, these recognition sites are built into the backbone of the oligomer and are fixed by the chemistry used to synthesise the molecules. We recently reported a different architecture, which is based on the nucleic acid blueprint in Fig. 1(a).^2^ If the recognition sites are appended as side chains on the backbone, it is possible to vary these functional groups independently of the rest of the molecule. Indeed a range of variants on nucleic acids have been prepared using the same backbone found in DNA but different types of base-pair.^3^ This approach therefore offers the potential for independent optimisation of the backbone, synthesis and recognition modules highlighted in Fig. 1(a).

Fig. 1(b) shows the structure of a duplex designed using this blueprint. The oligomers were prepared using reductive amination chemistry to generate a relatively non-polar backbone: there are no H-bond donors on the backbone, and the aniline nitrogen and aryl ether oxygen sites are very poor H-bond acceptors (*β* ≈ 4 and 3 respectively compared with 10 for the phosphine oxide).^4^ The recognition module is a single H-bond between a good H-bond donor (phenol, D), and a very good H-bond acceptor (phosphine oxide, A), which ensures efficient formation of duplexes with an increase of an order of magnitude in the association constant for every recognition unit added. We have recently shown that the three different backbone modules illustrated in Fig. 1(b) can be used interchangeably to form duplexes of comparable stability.^5^

**Fig. 2:**
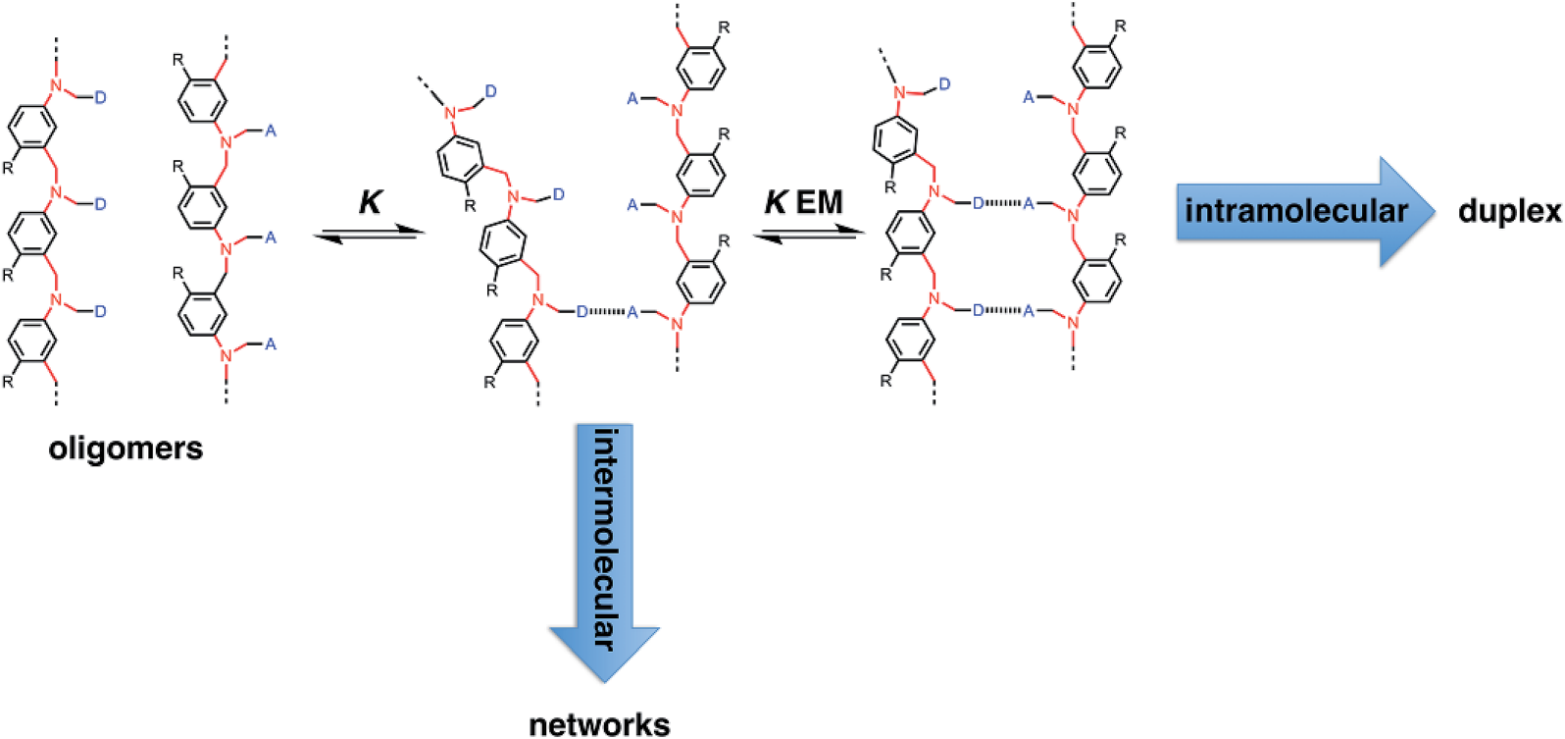
Stepwise assembly of a duplex from two complementary oligomers. There is an intermolecular channel that leads to cross-linked polymeric networks and an intramolecular channel that leads to duplex formation. *K* is the association constant for formation of an intermolecular interaction between two complementary H-bonding sites (blue bars), EM is the effective molarity for formation of an intramolecular interaction.

Fig. 2 illustrates the stepwise equilibria involved in assembly of a duplex. The efficiency of duplex formation is quantified by the parameter *K* EM, where *K* is the association constant for formation of an intermolecular A·D H-bond, and EM is the effective molarity for formation of an intramolecular H-bond.^6^ Cooperative formation of a duplex occurs if the product *K* EM is greater than one, because under these conditions, the intramolecular assembly channel shown in Fig. 2 is more favourable than the intermolecular channel that would lead to ill-defined aggregates (assuming the operating concentration *c* is less than EM). The values of EM for the six possible combinations of the three backbones shown in Fig. 1(b) are all in the range 7–20 mM, and the value of *K* for the phenol–phosphine oxide H-bond in toluene is about 300 M^–1^, so *K* EM is greater than one for all of these systems.^2^,^4^,^5^ Thus duplex formation appears to be rather insensitive to the conformational properties of the backbone, provided it has sufficient flexibility to accommodate simultaneous formation of H-bonds at multiple sites.

In this paper, we investigate the effects of changing the recognition module on duplex formation. The oligomers in Fig. 1(b) use a two-letter recognition alphabet, H-bond donor (D) and H-bond acceptor (A), and recognition is based on a single A·D H-bond. These oligomers should therefore be promiscuous: a H-bond donor oligomer can form a duplex with a variety of different H-bond acceptor oligomers. Here we compare the interactions of a family of H-bond donor oligomers with three different families of H-bond acceptor oligomer based on phosphine oxide, pyridine and pyridine N-oxide recognition modules.

## Results and discussion

### Synthesis

**Fig. 3:**
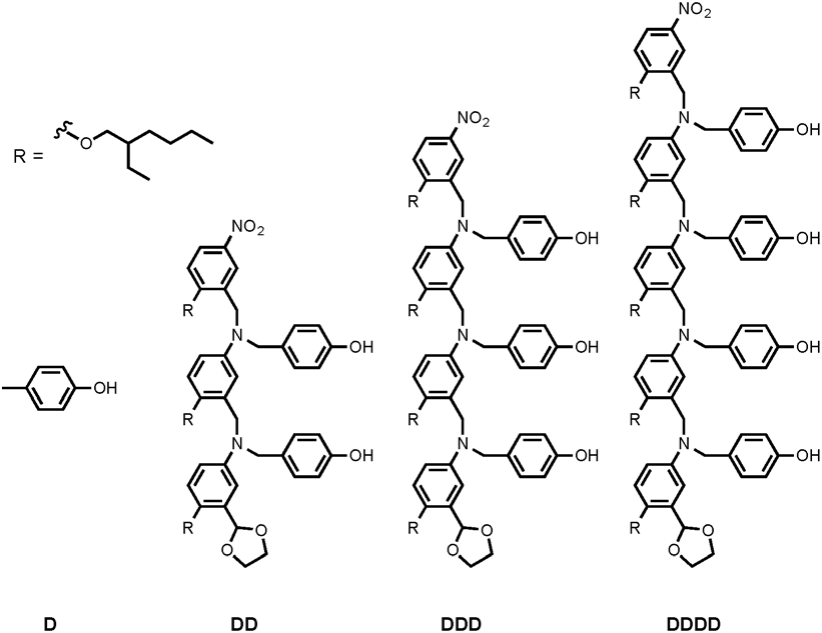
Structures of H-bond donor oligomers.

**Scheme 1:**
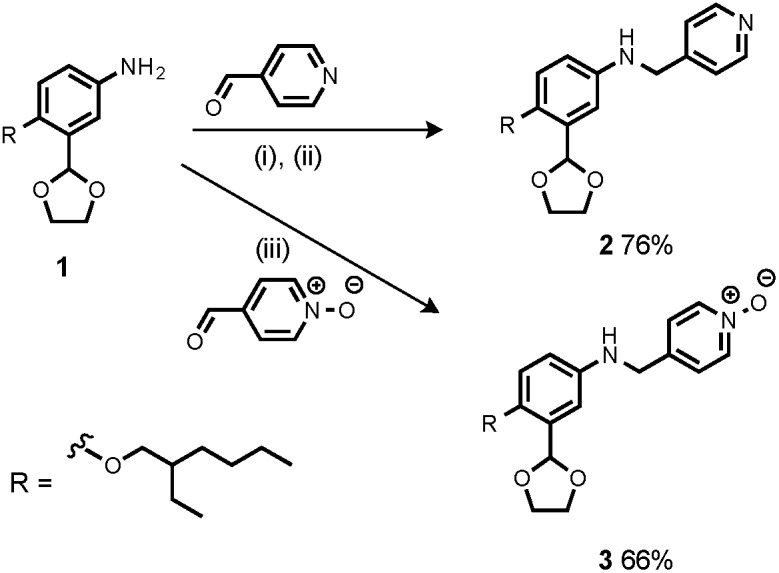
(i) Heat; (ii) NaBH_4_; (iii) NaBH(OAc)_3_.

Synthesis of the H-bond donor oligomers, DD, DDD and DDDD, has been reported previously (Fig. 3).^2^ The monomer units used for synthesis of the H-bond acceptor oligomers were prepared by coupling the relevant benzaldehyde derivative with aniline **1** (Scheme 1). The pyridine monomer **2** was prepared by reducing the imine formed between **1** and 4-nicotinaldehyde using NaBH_4_. The pyridine N-oxide monomer **3** was made by reductive amination of **1** and 4-formylpyridine-N-oxide using NaBH(OAc)_3_.^7^

**Scheme 2:**
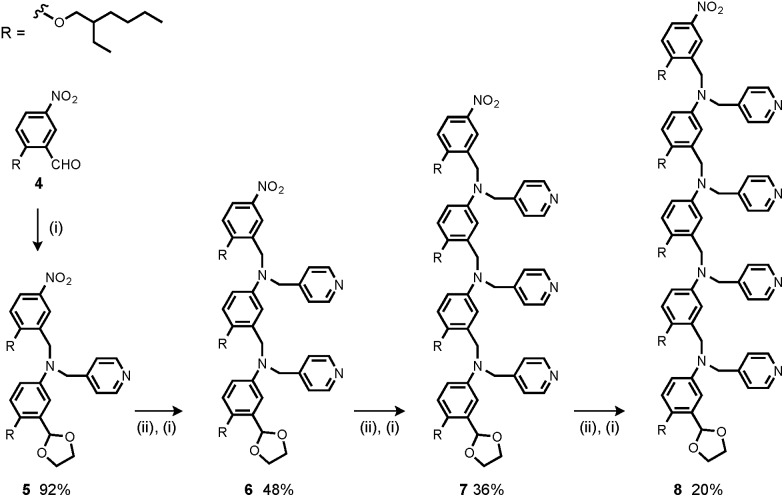
(i) **2**, NaBH(OAc)_3_; (ii) HCl.

**Scheme 3:**
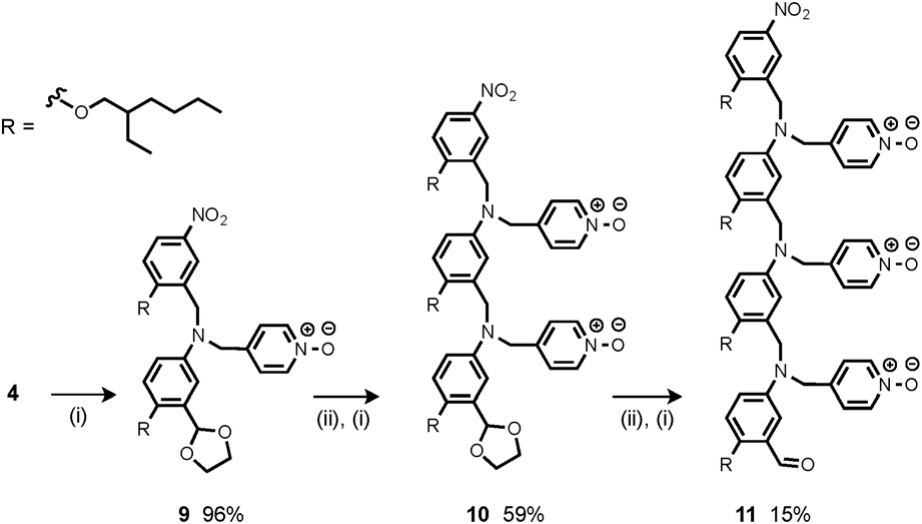
(i) **3**, NaBH(OAc)_3_; (ii) HCl.

**Scheme 4:**
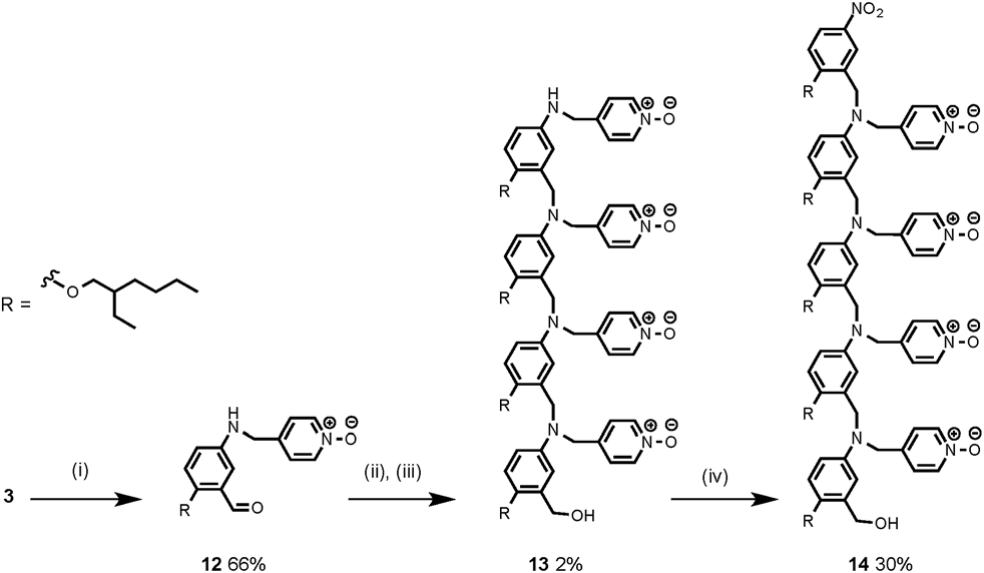
(i) aq. HCl; (ii) NaBH(AcO)_3_; (iii) NaBH_4_; (iv) **4**, NaBH(AcO)_3_.

The synthesis of **4** has been previously described, and here it was used as the starting point for growing the H-bond acceptor oligomer chains (Scheme 2).^2^ Iterative reductive amination and acetal deprotection steps were used to synthesise the three pyridine oligomers shown in Scheme 2 (**6**, **7** and **8**). Similarly, the pyridine N-oxide oligomers 2-mer and 3-mer were synthesised by sequential deprotection and coupling steps (Scheme 3). The pyridine N-oxide 3-mer was isolated as the aldehyde rather than the acetal (**11**). The pyridine N-oxide 4-mer proved difficult to obtain by this route and was therefore prepared by direct oligomerisation of compound **12** (Scheme 4). Reverse phase chromatography was used to separate the mixture of oligomers obtained under reductive amination conditions. Compound **13** was isolated from this mixture, and reductive amination with **4** gave the pyridine N-oxide 4-mer with an alcohol as the terminal functional group (**14**). Acetals, aldehydes and alcohols are all much weaker H-bond acceptors than pyridine N-oxides, so differences in the nature of the terminal functional groups in the pyridine N-oxide oligomers do not significantly affect the duplex assembly properties of these systems.

### NMR binding studies

The association constants of length-complementary duplexes were measured by fitting ^1^H NMR titration data in toluene-d8 to a 1 : 1 binding isotherm (see ESI for details†). Association constants for the corresponding 1-mer complexes, A·D, were measured by titrating 4-methylpyridine or 4-methylpyridine N-oxide into 4-methylphenol in toluene-d8. The association constants for duplex formation are provided in Table 1, along with values of EM and *K* EM calculated using eqn (1).

1 *K*_*N*_ = 2*K*^*N*^EM^*N*–1^

where *K*_*N*_ is the association constant for a duplex with *N* recognition units, *K* is the association constant for the A·D complex that makes a single H-bond, and EM is the effective molarity for formation of intramolecular H-bonds in the duplex (assuming that all stepwise intramolecular interactions shown in Fig. 2 have the same EM).

**Table 1:** Association constants (*K*_*N*_) and effective molarities (EM) for formation of 1 : 1 duplexes measured using ^1^H NMR titrations in toluene at 298 K^*a*^

Complex	log *K*_*N*_/M^–1^	EM/mM	*K* EM
**Pyridine oligomers**			
A·D	1.5 ± 0.1	—	—
AA·DD	2.1 ± 0.1	57 ± 8	2 ± 1
AAA·DDD	2.7 ± 0.1	82 ± 6	3 ± 1
AAAA·DDDD	3.6 ± 0.3	110 ± 30	4 ± 1

**Pyridine N-oxide oligomers**			
A·D	2.5 ± 0.1	—	—
AA·DD	3.7 ± 0.1	30 ± 10	8 ± 4
AAA·DDD	5.0 ± 0.2	40 ± 10	12 ± 5
AAAA·DDDD	6.6 ± 0.2	60 ± 10	18 ± 6

^*a*^Each titration was repeated twice and the average value is reported with errors at the 95% confidence limit.

For both the pyridine and pyridine N-oxide systems, there is a uniform increase in the stabilities of the complexes with increasing numbers of recognition units, which is indicative of cooperative H-bonding along the oligomers and fully assembled duplex formation. This conclusion is consistent with the values of *K* EM, which are greater than one in all cases, indicating that intramolecular H-bonding is favoured over the intermolecular interactions that would lead to higher order complexes. The values of EM are almost independent of the length of the oligomer for both types of H-bond acceptor, which implies that there is good geometric complementarity between the two strands of the duplex in both systems. Although the limiting complexation-induced changes in ^1^H NMR chemical shift are small, there are similar patterns of chemical shift change within the two families of duplex, which suggests that they have similar structures (see ESI†). For example, the signals due to the pyridine alpha protons all show an upfield shift about 0.2 ppm on duplex formation, and the signals due to the CH_2_ groups of the nitrobenzyl moieties all show a small downfield shift of about 0.05 ppm.

**Fig. 4:**
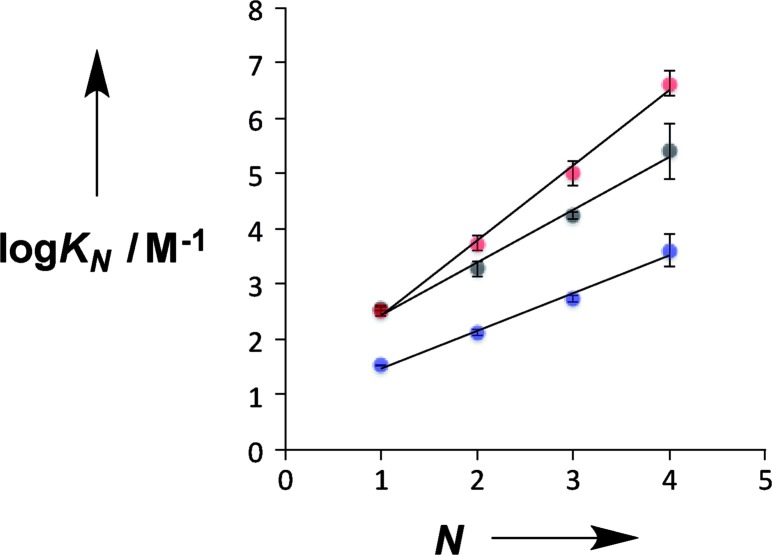
Relationship between the association constant for duplex formation (log *K*_*N*_) and the number of recognition modules in an oligomer (*N*). The H-bond acceptor modules are pyridine N-oxide (red), pyridine (blue) and phosphine oxide (black). The lines of best fit are shown for each type of duplex: pyridine N-oxide, log *K*_*N*_ = 1.4*N* + 1.1; pyridine, log *K*_*N*_ = 0.7*N* + 0.8; phosphine oxide, log *K*_*N*_ = 1.0*N* + 1.5.

Fig. 4 compares the properties of pyridine and pyridine N-oxide duplexes with the phosphine oxide duplexes previously reported.^2^ For all three systems, the logarithm of the association constant for duplex formation (log *K*_*N*_) increases linearly with the number of recognition units, *N*. For the phosphine oxide system, the association constant increases by an order of magnitude for each additional H-bond (the slope of the correlation in Fig. 4 is 1.0). The slope of the correlation in Fig. 4 is significantly larger for the pyridine N-oxide duplexes (1.4) and significantly lower for the pyridine duplexes (0.7).

**Fig. 5:**
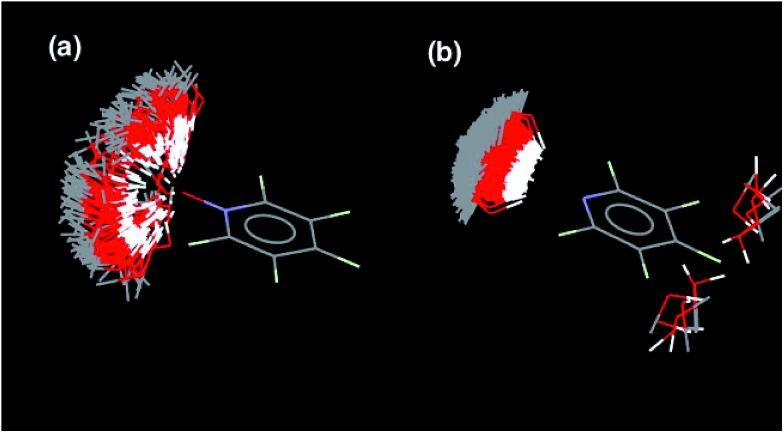
Distribution of phenol OH groups around (a) pyridine N-oxide H-bond acceptors and (b) pyridine H-bond acceptors in the CSD. Data from IsoStar 2.2.3 for contacts that are closer than the sum of the van der Waals radii and plotted using the symmetry-expanded display. For the phenol–pyridine system, there are a small number of examples cases (right) that do not correspond to H-bonding interactions.

There are two factors that contribute to the relationship between log *K*_*N*_ and *N*: the value of EM for formation of intramolecular H-bonds, and the intrinsic strength of the H-bonding interactions, which is quantified by the value of *K* for the 1-mer A·D complex. The phosphine oxide and pyridine N-oxide oligomers make H-bonds of a similar strength (log *K* = 2.5), but the average EM for formation of the pyridine N-oxide duplexes (40 mM) is significantly larger than the average EM for formation of the phosphine oxide duplexes (14 mM). This result can be explained by the fact that the phosphine oxide recognition modules have more conformational degrees of freedom than the pyridine N-oxide modules. In general, more rigid and preorganised structures lead to higher values of EM.^8^,^9^ The average EM for the pyridine duplexes is even higher (80 mM). Although the pyridine and pyridine N-oxide recognition modules share the same basic scaffold, the phenol–pyridine H-bond is more directional than the phenol–pyridine N-oxide H-bond, which in effect has an additional degree of freedom through variation in the H-bond geometry. Fig. 5 shows the distribution of phenol–pyridine N-oxide and phenol–pyridine H-bonding interactions found in small molecule X-ray crystal structures in the Cambridge Structural Database (CSD).^10^ The interactions with pyridine all occur at a very well-defined location along the nitrogen lone pair direction (Fig. 5(b)). In contrast, the interactions with pyridine N-oxide sample a wide variety of different geometries (Fig. 5(a)).

Thus the pyridine duplexes are the most highly organised complexes and have the largest EM, but the intrinsic strength of the pyridine–phenol H-bond is an order of magnitude weaker than the H-bonds in the other two types of duplex (log *K* = 1.5), and so the overall increase in stability with *N* is smaller for the pyridine duplexes: the average *K* EM is 3 compared with 5 and 13 for the phosphine oxide and pyridine N-oxide duplexes respectively. For all three types of duplex, there is a small but consistent increase in EM with *N*, which might suggest some additional cooperativity due to nucleation of a more highly organised duplex structure as the chains grow longer. However, the increases in EM are close to the error margins, so it is difficult to draw any definite conclusions.

## Conclusions

If two oligomeric chains are functionalised with complementary recognition sites, they will interact to form a duplex provided the product *K* EM is greater than one: *K* is the association constant for a single intermolecular interaction between two complementary recognition sites and depends on the nature of the functional groups involved; EM is the effective molarity for intramolecular interactions that lead to zipping up of the duplex and depends on the geometric complementarity and complementarity of the backbone chains. In previous work, we have shown that duplex formation is tolerant of changes in the backbone, which lead to rather small variations in EM (7–20 mM). In this paper, we have investigated changes in the recognition modules. H-Bond donor oligomers bearing phenol recognition groups form stable duplexes with three different types of H-bond acceptor oligomer bearing phosphine oxide, pyridine or pyridine N-oxide recognition groups. In all three cases, the stability of the duplexes increase with increasing numbers of recognition sites in the oligomers indicating cooperative duplex assembly. However, the different recognition modules are found to affect EM as well as *K*. Phenol–pyridine N-oxide and phenol–phosphine oxide H-bonds are both an order of magnitude stronger than phenol–pyridine H-bonds in toluene, and the stronger interactions lead to more stable duplexes. Due to differences in conformational flexibility, the EM for duplex assembly is greater for the pyridine oligomers (80 mM) than for the pyridine N-oxide oligomers (40 mM), which in turn have a greater EM than the phosphine oxide oligomers (14 mM). As a result, the pyridine N-oxide oligomers form the most stable duplexes, due a combination of high *K* and high EM. These systems demonstrate that it is possible mix and match both the backbone and the recognition modules in synthetic H-bonded duplexes making them particularly versatile and robust supramolecular assembly motifs. The use of a single donor–acceptor H-bond as the recognition element provides these systems an unusual degree of promiscuity making it possible to switch between different donor–acceptor recognition alphabets.

## Supplementary Material

Supplementary information
