# Supplementary material for: Mix and match recognition modules for the formation of H-bonded duplexes
Source: Chem Sci. 2016 Jun 7;7(9):5686–91. doi: 10.1039/c6sc01884j (PMC6022071; doi:10.1039/c6sc01884j)
Supplement: Supplementary file 1 [file SC-007-C6SC01884J-s001.pdf]

Electronic Supplementary Material (ESI) for Chemical Science.
This journal is © The Royal Society of Chemistry 2016

Supporting information

Mix and Match Recognition Modules for the Formation of H-Bonded Duplexes

Alexander E. Stross, Giulia Iadevaia, Christopher A. Hunter*

Department of Chemistry, University of Cambridge, Lensfield Road, Cambridge CB2 1EW (UK)

Table of Contents

Synthesis S2

NMR titrations S30

Synthesis

Synthesis of 2

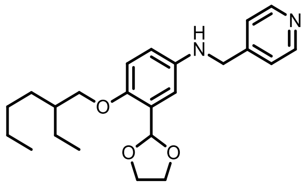

1 (11.5 g, 39.3 mmol, 1 equiv.) and 4-nicotinaldehyde (4.1 mL, 43.2 mmol, 1.1 equiv.) were dissolved in $CHCl_3$ (80 mL) in the presence of molecular sieves with stirring. After 6 hours the solution was filtered and the solvent removed by rotary evaporator. This crude mixture was dissolved in MeOH (150 mL) and then $NaBH_4$ (4.45 g, 118 mmol, 3 equiv.) was added slowly at 0 °C with stirring. This mixture was stirred for 2 hours before the solution was neutralized using concentrated aqueous HCl. This solution was washed with EtOAc (5 × 20 mL) and all the organic extracts were combined and washed with brine (1 × 20 mL) and then dried ($MgSO_4$). The crude oil was purified via flash chromatography on silica eluting with a gradient from 75% to 100% EtOAc in hexane to yield a golden oil (11.4 g, 76%).

^{1}H NMR (250 MHz, $CDCl_3$): δ_H = 8.53 (d, 2H, *J* = 6.0), 7.28 (d, 2H, *J* = 6.0), 6.85 (d, 1H, *J* = 3.0), 6.74 (d, 1H, *J* = 9.0), 6.48 (dd, 1H, *J* = 9.0, *J* = 3.0), 6.11 (s, 1H), 4.33 (s, 2H), 3.96 – 4.10 (m, 4H), 3.79 (d, 2H, *J* = 5.5), 1.67 – 1.77 (m, 1H), 1.24 – 1.54 (m, 8H), 0.86 – 0.95 (m, 6H);

^{13}C NMR (101 MHz, $CDCl_3$): δ_C = 150.4, 150.0, 149.2, 141.4, 127.2, 122.2, 114.1, 113.8, 112.2, 99.2, 71.9, 65.2, 47.9, 39.5, 30.5, 29.1, 23.9, 23.1, 14.1, 11.1;

MS (ES+): m/z (%) = 385 (100) [M + H$^+$], 426 (40);

HRMS (ES+): calculated for $C_{23}H_{33}N_2O_3$ 385.2491, found 385.2494;

FT-IR (ATR): ν_{max}/cm^{-1} 2957, 2926, 2859, 1680, 1603, 1496, 1465, 1388, 1253, 1224, 1174, 1068, 1030.

Synthesis of 3

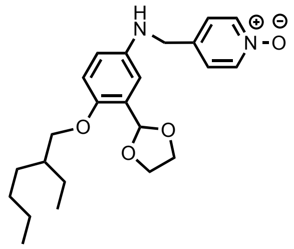

1 (1.008 g, 3.44 mmol, 1 equiv.) and 4-formylpyridine-*N*-oxide (0.508 g, 4.12 mmol, 1.2 equiv.) were dissolved in $CHCl_3$ (12 mL) with stirring and $NaBH(OAc)_3$ (2.04 g, 9.62 mmol, 2.8 equiv.) was added at room temperature. After 1 h the reaction was quenched with saturated aq. $NaHCO_3$ solution, and extracted into $CHCl_3$ (4 × 10 mL). All the organic fractions were washed with water (1 × 10 mL), brine (1 × 10 mL) and dried ($MgSO_4$) before the solvent removed using a rotary evaporator. The crude product was purified using flash chromatography on silica eluting with a gradient from 0 to 8% of MeOH in $CHCl_3$ to yield a yellow powder (0.91 g, 66%)

^{1}H NMR (400 MHz, $CDCl_3$): δ_H = 8.16 (d, 2H, *J* = 7.0), 7.29 (d, 2H, *J* = 7.0), 6.85 (d, 1H, *J* = 3.0), 6.75 (d, 1H, *J* = 9.0), 6.48 (dd, 1H, *J* = 9.0, *J* = 3.0), 6.08 (s, 1H), 4.32 (s, 2H), 4.13 – 3.96 (m, 4H), 3.80 (d, 2H, *J* = 6,), 1.76 – 1.66 (m, 1H), 1.55 – 1.23 (m, 8H), 0.94 – 0.85 (m, 6H);

^{13}C NMR (101 MHz, $CDCl_3$): δ_C = 150.7, 141.0, 140.1, 139.2, 127.3, 124.6, 114.3, 113.9, 112.3, 99.2, 72.0, 65.3, 47.2, 39.6, 30.6, 29.1, 24.0, 23.2, 14.2, 11.2;

HRMS (ES+): calculated for $C_{23}H_{33}N_2O_4$ 401.2440, found 401.2435;

FT-IR (thin film): ν_{max}/cm^{-1} 3055, 2987, 2686, 2411, 2306, 1505,1483, 1422, 1265, 1168, 1071, 896.

M.p. 126 – 134 °C.

Synthesis of 5

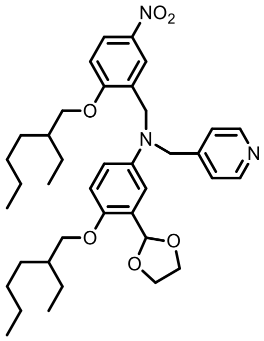

2 (0.913 g, 2.37 mmol, 1 equiv.) and **4** (1.061 g, 3.80 mmol, 1.6 equiv.) were dissolved in DCE (8.5 mL) and $NaBH(OAc)_3$ (1.41 g, 6.6 mmol, 2.8 equiv.) was added with stirring. After 18 hours the reaction was quenched with saturated aqueous $NaHCO_3$ solution, and extracted into $CHCl_3$ (4 × 10 mL). All the organic fractions were washed with water (1 × 10 mL), brine (1 × 10 mL) and dried ($MgSO_4$) before the solvent removed with a rotary evaporator. The crude product was purified using flash chromatography on silica eluting with a gradient from 0% to 50% of EtOAc in hexane to yield a golden yellow oil (1.4 g, 92%).

^{1}H NMR (400 MHz, $CDCl_3$): δ_H = 8.53 (d, 2H, *J* = 6.0), 8.15 (dd, 1H, *J* = 9.0, *J* = 3.0), 8.11 (d, 1H, *J* = 3.0), 7.21 (d, 2H, *J* = 6.0), 6.93 (d, 1H, *J* = 9.0), 6.91 (d, 1H, *J* = 3.0), 6.75 (d, 1H, *J* = 9.0), 6.57 (dd, 1H, *J* = 9.0), 6.10 (s, 1H), 4.62 (s, 2H), 4.55 (s, 2H), 3.98 (d, 2H, *J* = 6.0), 3.93 (s, 4H), 3.80 (d, 2H, *J* = 6.0), 1.66 – 1.83 (m, 2H), 1.23 – 1.53 (m, 16H), 0.83 – 0.96 (m, 12H);

^{13}C NMR (101 MHz, $CDCl_3$): δ_C = 161.8, 150.4, 149.9, 148.4, 142.1, 141.4, 128.0, 127.1, 124.7, 123.4, 122.1, 114.8, 113.5, 112.2, 110.5, 99.4, 71.5, 65.0, 54.6, 50.6, 39.4, 39.1, 30.5, 29.0, 23.9, 23.0, 22.9, 14.0, 11.1;

MS (ES+): m/z (%) = 648 (100) [M + H^+], 689 (30) [MH^+ + CH_3CN];

HRMS (ES+): calculated for $C_{38}H_{54}N_3O_6$ 648.4013, found 648.4016;

FT-IR (thin film): ν_{max}/cm^{-1} 2958, 2927, 2859, 1681, 1592, 1501, 1463, 1338, 1264, 1230, 1179, 1074, 1014.

Synthesis of 5'

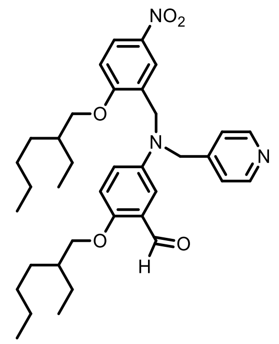

5 (1.51 g, 2.3 mmol, 1 equiv.) was dissolved in $CHCl_3$ (10 mL) and concentrated aqueous HCl (10 mL) was added with stirring. After 18 hours the mixture was neutralised using aqueous $NaHCO_3$ and the organic portion separated from the aqueous part. The aqueous layer was washed with $CHCl_3$ (3 × 10 mL) before all organic fractions were washed with brine (1 × 10 mL) dried ($MgSO_4$) and the solvent removed with a rotary evaporator to yield an intense yellow oil (1.38 g, 98%) requiring no further purification.

^{1}H NMR (500 MHz, $CDCl_3$): δ_H = 10.44 (s, 1H), 8.54 (d, 1H, *J* = 6.0), 8.15 (dd, 1H, *J* = 9.0), 8.00 (d, 1H, *J* = 3.0), 7.18 (d, 2H, *J* = 6.0), 7.13 (d, 1H, *J* = 2.5), 6.95 (d, 1H, *J* = 9.0), 6.87 (d, 1H, *J* = 9.0), 6.84 (dd, 1H, *J* = 9.0, *J* = 3.0), 4.64 (s, 2H), 4.59 (s, 2H), 4.00 (d, 2H, *J* = 5.5), 3.85 – 3.91 (m, 2H), 1.69 – 1.82 (m, 2H), 1.23 – 1.53 (m, 16H), 0.84 – 0.96 (m, 12H);

^{13}C NMR (126 MHz, $CDCl_3$): δ_C = 189.7, 161.9, 155.1, 150.0, 147.6, 142.1, 141.3, 127.2, 125.3, 124.8, 122.9, 121.8, 120.8, 114.3, 110.9, 110.6, 71.6, 71.2, 54.2, 50.2, 39.5, 39.1, 30.6, 30.4, 29.0, 28.9, 23.9, 23.8, 22.9, 22.8, 14.0, 13.9, 11.1, 11.0;

MS (ES+): m/z (%) = 604 (100) [M + H^+], 645 (50) [MH^+ + CH_3CN];

HRMS (ES+): calculated for $C_{36}H_{50}N_3O_5$ 604.3750, found 604.3740;

FT-IR (thin film): ν_{max}/cm^{-1} 2957, 2923, 2855, 1680, 1598, 1501, 1464, 1339, 1265, 1228, 1180, 1015.

Synthesis of 6

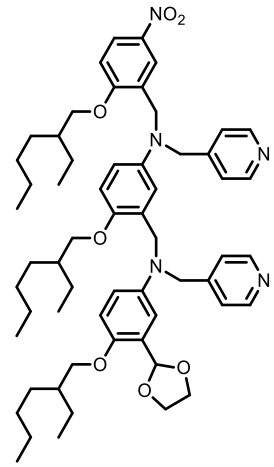

5' (0.913 g, 2.37 mmol, 1 equiv.) and **2** (1.061 g, 3.80 mmol, 1.6 equiv.) were dissolved in DCE (8.5 mL) and $NaBH(OAc)_3$ (1.41 g, 6.6 mmol, 2.8 equiv.) was added with stirring. After 18 hours the reaction was quenched with saturated aqueous $NaHCO_3$ solution, and extracted into $CHCl_3$ (4 × 10 mL). All the organic fractions were washed with water (1 × 10 mL), brine (1 × 10 mL) and dried ($MgSO_4$) before the solvent removed with a rotary evaporator. The crude product was purified using flash chromatography on silica eluting with a gradient from 0% to 50% of EtOAc in hexane to yield a golden yellow oil (1.4 g, 92%).

^{1}H NMR (500 MHz, $CDCl_3$): δ_H = 8.51 (d, 2H, *J* = 6.0), 8.47 (d, 2H, *J* = 6.0), 8.07 (dd, 1H, *J* = 9.0, 3.0), 7.94 (d, 1H, *J* = 3.0), 7.12 – 7.08 (m, 4H), 6.84 (d, 1H, *J* = 9.0), 6.72 (d, 1H, *J* = 9.0), 6.68 (d, 1H, *J* = 3.0), 6.59 (d, 1H, *J* = 9.0), 6.46 (dd, 1H, *J* = 9.0, 3.0), 6.37 – 6.33 (m, 2H), 6.07 (s, 1H), 4.53 – 4.49 (m, 4H), 4.47 (s, 2H), 4.18 (s, 2H), 3.94 (d, 2H, *J* = 6.0), 3.93 – 3.90 (m, 4H), 3.80 (d, 2H, *J* = 6.0), 3.76 (d, 2H, *J* = 5.5), 1.80 – 1.70 (m, 2H), 1.68 – 1.61 (m, 1H), 1.57 – 1.19 (m, 24H), 0.98 – 0.81 (m, 18H);

^{13}C NMR (126 MHz, $CDCl_3$): δ_C = 161.5, 149.8, 149.7, 149.6, 149.4, 148.7, 142.4, 141.6, 141.2, 127.6, 126.8, 126.6, 124.5, 122.8, 121.9, 113.9, 113.4, 113.0, 112.3, 111.6,

111.2, 110.6, 99.3, 71.7, 71.4, 70.7, 65.0, 55.1, 53.7, 50.8, 50.0, 39.5, 39.4, 39.2, 30.6, 30.5, 29.1, 29.0, 24.0, 23.9, 23.9, 23.0, 22.9, 22.9, 14.0, 14.0, 11.1, 11.1;;

MS (ES+): m/z (%) = 487 (20), 507 (80), 973 (100) [M + H^+], 995 (30) [M + Na^+];

HRMS (ES+): calculated for $C_{59}H_{82}N_5O_7$ 972.6214, found 972.6240;

FT-IR (thin film): ν_{max}/cm^{-1} 2958, 2928, 2872, 1681, 1599, 1504, 1465, 1340, 1266, 1228, 1066, 1026.

Synthesis of 6'

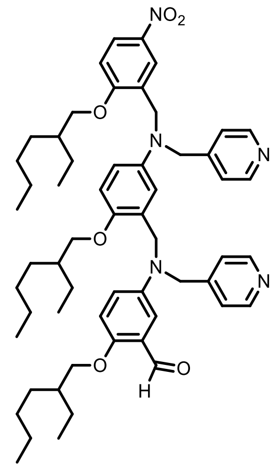

6 (1.51 g, 2.3 mmol, 1 equiv.) was dissolved in $CHCl_3$ (10 mL) and concentrated aqueous HCl (10 mL) was added with stirring. After 18 hours the mixture was neutralised using aqueous $NaHCO_3$ and the organic portion separated from the aqueous part. The aqueous layer was washed with $CHCl_3$ (3 × 10 mL) before all organic fractions were washed with brine (1 × 10 mL) dried ($MgSO_4$) and the solvent removed with a rotary evaporator to yield an intense yellow oil (1.38 g, 98%) requiring no further purification.

^{1}H NMR (400 MHz, $CDCl_3$): δ_H = 10.42 (s, 1H), 8.53 – 8.47 (m, 4H), 8.05 (dd, 1H, *J* = 9.0, 3.0), 7.90 (d, 1H, *J* = 3.0), 7.11 (d, 2H, *J* = 6.0) 7.07 (d, 2H, *J* = 6.0), 6.89 (d, 1H, *J* = 3.0), 6.80 (d, 1H, *J* = 9.0), 6.73 (d, 1H, *J* = 9.0), 6.67 (d, 1H, *J* = 9.0), 6.59 (dd, 1H, *J* = 9.0, 3.0), 6.47 (dd, 1H, *J* = 9.0, 3.0), 6.27 (d, 1H, *J* = 3.0), 4.54 (s, 2H), 4.48 (s, 4H), 4.27 (s, 2H), 3.91 (d, 2H, *J* = 6.0), 3.88 (d, 2H *J* = 6.0), 3.78 (d, 2H, *J* = 6.0), 1.82 – 1.71 (m, 2H), 1.71 – 1.61 (m, 1H), 1.59 – 1.19 (m, 24H), 1.01 – 0.81 (m, 18H);

^{13}C NMR (101 MHz, $CDCl_3$): δ_C = 189.9, 161.5, 154.3, 149.9, 149.7, 149.6, 148.4, 148.3, 142.2, 141.7, 141.2, 127.6, 126.1, 125.0, 124.5, 122.8, 121.8, 121.7, 120.2, 113.9, 112.6, 112.4, 111.7, 110.4, 109.6, 71.5, 71.3, 70.7, 55.0, 53.6, 50.9, 49.9, 39.6, 39.4, 39.2, 30.6, 30.6, 30.5, 29.1, 29.0, 29.0, 24.0, 24.0, 23.9, 23.0, 23.0, 22.9, 14.1, 14.0, 11.2, 11.1;

MS (ES+): m/z (%) = 485 (85) , 506 (50), 929 (100) [M + H^+], 951 (40) [M + Na^+];

HRMS (ES+): calculated for $C_{57}H_{78}N_5O_6$ 928.5952, found 928.5937;

FT-IR (thin film): ν_{max}/cm^{-1} 2957, 2926, 2858, 1680, 1598, 1500, 1463, 1414, 1338, 1264, 1226, 1179, 1015, 969.

Synthesis of 7

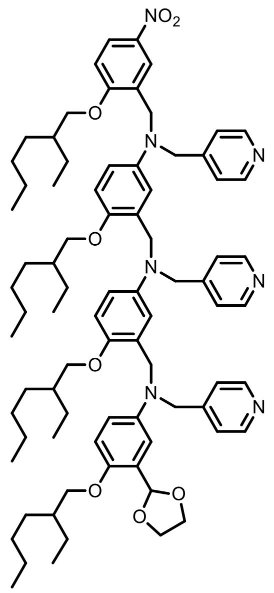

2 (0.0519 g, 0.13 mmol, 1 equiv.) and **6'** (0.188 g, 0.200 mmol, 1.5 equiv.) were dissolved in DCE (0.5 mL) and $NaBH(OAc)_3$ (0.080 g, 0.38 mmol, 2.8 equiv.) was added with stirring. After 3 days the reaction was quenched with saturated aqueous $NaHCO_3$ solution, and extracted into $CHCl_3$ (4 × 10 mL). All the organic fractions were washed with water (1 × 10 mL), brine (1 × 10 mL) and dried ($MgSO_4$) before the solvent removed on a rotary evaporator. The crude product was purified using flash chromatography on silica eluting with a gradient from 80% to 100% of EtOAc (with 1% NEt_3) in hexane (with 1% NEt_3 to yield a golden yellow oil (0.062 g, 36%).

^{1}H NMR (400 MHz, $CDCl_3$): δ_H = 8.40 – 8.46 (m, 6H), 8.08 (dd, 1H, *J* = 9.0, *J* = 3.0), 7.97 (d, 1H, *J* = 3.0), 7.07 (d, 2H, *J* = 6.0), 7.02 (d, 2H, *J* = 6.0), 6.97 (d, 2H, *J* = 6.0), 6.86 (d, 1H, *J* = 9.0), 6.78 (d, 1H, *J* = 3.0), 6.71 (d, 1H, *J* = 8.5), 6.68 (d, 1H, *J* = 9.0), 6.62 (d, 1H, *J* = 9.0), 6.39 – 6.47 (m, 3H), 6.34 (dd, 1H, *J* = 9.0, 2.9), 6.26 (d, 1H, *J* = 3.0), 6.09 (s, 1H), 4.51 (s, 2H), 4.42 – 4.48 (m, 6H), 4.02 – 4.07 (m, 4H), 3.89 – 3.95 (m, 6H), 3.77 – 3.81 (m, 4H), 3.75 (d, 2H, *J* = 6.0), 1.59 – 1.79 (m, 4H), 1.16 – 1.54 (m, 36H), 0.77 - 0.97 (m, 24H);

^{13}C NMR (63 MHz, $CDCl_3$): δ_C = 161.6, 149.8, 149.5, 149.3, 149.2, 149.1, 148.5, 142.5, 142.2, 141.7, 141.3, 127.9, 127.1, 126.8, 126.5, 124.6, 123.0, 122.0, 114.4, 113.6, 113.1, 112.8, 112.4, 112.3, 111.9, 111.8, 110.5, 99.4, 71.8, 71.5, 70.8, 70.7, 65.0, 54.9, 54.1, 53.8, 51.1, 50.9, 50.2, 39.5, 39.2, 30.7, 30.6, 30.5, 29.7, 29.1, 29.0, 24.0, 23.9, 23.1, 23.0, 22.9, 14.1, 14.0, 11.2, 11.1;

MS (ES+): m/z (%) = 274 (60), 649 (20), 1253 (10), 1297 (100) [M + H^+];

HRMS (ES+): calculated for $C_{80}H_{110}N_7O_8$ 1296.8416, found 1296.8459;

FT-IR (thin film): ν_{max}/cm^{-1} 2957, 2926, 2874, 2859, 1679, 1599, 1502, 1463, 1414, 1339, 1264, 1226, 1180, 1065, 1028, 969.

Synthesis of 7'

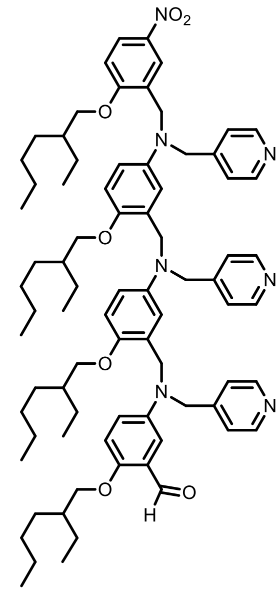

7 (0.25 g, 0.19 mmol, 1 equiv.) was dissolved in $CHCl_3$ (10 mL) and concentrated aqueous HCl (10 mL) was added with stirring. After 2 days the mixture was neutralised using aqueous $NaHCO_3$ and the organic portion separated from the aqueous part. The aqueous layer was washed with $CHCl_3$ (3 × 10 mL) before all organic fractions were washed with brine (1 × 10 mL) dried ($MgSO_4$) and the solvent removed with a rotary evaporator to yield a yellow oil (0.22 g, 90%) requiring no further purification.

^{1}H NMR (400 MHz, CDCl$_3$): δ_H = 10.42 (s, 1H), 8.44 (d, 2H, *J* = 6.0), 8.40 (d, 4H, *J* = 6.0), 8.06 (dd, 1H, *J* = 9.0, *J* = 3.0), 7.97 (d, 1H, *J* = 2.5), 7.02 (d, 4H, *J* = 5.5), 6.94 (d, 2H, *J* = 6.0), 6.87 (d, 1H, *J* = 9.0), 6.71 – 6.76 (m, 2H), 6.68 (d, 1H, *J* = 9.0), 6.59 – 6.66 (m, 2H), 6.45 (dd, 1H, *J* = 9.0, *J* = 3.0), 6.39 (d, 1H, *J* = 2.5), 6.32 (dd, 1H, *J* = 9.0, *J* = 3.0), 6.17 (d, 1H, *J* = 3.0), 4.51 (s, 2H), 4.47 (s., 2H), 4.46 (s, 2H), 4.43 (s, 2H), 4.10 (s, 2H), 4.08 (s, 2H), 3.93 (d, 2H, *J* = 5.5), 3.86 (d, 2H. *J* = 5.5), 3.79 (d, 2H, *J* = 5.5), 3.71 (d, 2H, *J* = 5.5), 1.55 – 1.79 (m, 4H), 1.17 – 1.54 (m, 32H), 0.76 – 0.96 (m, 24H);

^{13}C NMR (63 MHz, CDCl$_3$): δ_C = 189.5 ,161.4, 154.1, 149.7, 149.6, 149.3, 148.8, 148.7, 148.1, 148.0, 142.4, 141.9, 141.6, 141.1, 127.8, 126.8, 125.5, 124.9, 124.3, 122.8, 121.6, 121.5, 121.4, 120.3, 113.9, 112.8, 112.2, 112.1, 111.5, 110.3, 110.1, 71.3, 71.1, 70.5, 54.7,

54.1, 53.1, 50.8, 50.7, 49.9, 39.3, 39.2, 39.0, 30.5, 30.4, 30.3, 28.9, 28.8, 28.7, 23.8, 23.7, 22.8, 22.7, 13.9, 13.8, 13.7, 11.0, 10.9;

MS (ES+): m/z (%) = 1252 (50), 1253 (100) [M + H^+], 1254 (60), 1255 (20);

HRMS (ES+): calculated for $C_{78}H_{106}N_7O_7$ 1252.8154, found 1252.8148;

FT-IR (thin film): ν_{max}/cm^{-1} 2957, 2922, 2854, 1681, 1599, 1502, 1464, 1339, 1265, 1226, 1180.

Synthesis of 8

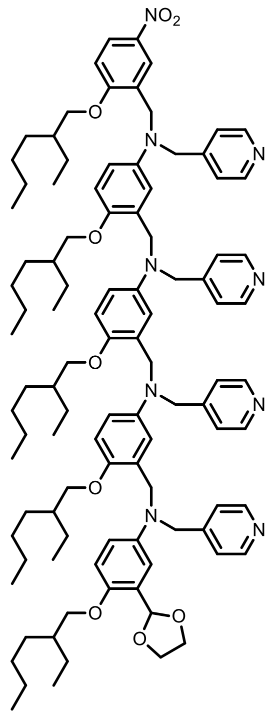

7' (0.20 g, 0.16 mmol, 1 equiv.) and **2** (0.12 g, 0.31 mmol, 2 equiv.) were dissolved in DCE (1.2 mL) and $NaBH(OAc)_3$ (0.093 g, 0.44 mmol, 2.8 equiv.) was added with stirring. After 3 days the reaction was quenched with saturated aqueous $NaHCO_3$ solution, and extracted into $CHCl_3$ (4 × 10 mL). All the organic fractions were washed with water (1 × 10 mL), brine (1 × 10 mL) and dried ($MgSO_4$) before the solvent removed with a rotary evaporator. The crude product was purified using flash chromatography on silica eluting with a gradient from 0% to 10% of MeOH in $CHCl_3$ and then on reverse-phase silica eluting with a gradient from 70% to 100% of MeOH in acetonitrile) to yield a golden yellow oil (0.051 g, 20%).

^{1}H NMR (400 MHz, $CDCl_3$): δ_H = 8.44 (d, 2H, *J* = 6.0), 8.39 – 8.42 (m, 4H), 8.31 (d, 1H, *J* = 6.0), 8.09 (dd, 1H, *J* = 9.0, *J* = 3.0), 7.98 (d, 1H, *J* = 3.0), 7.03 (d, 2H, *J* = 6.0), 7.00 (d, 2H, *J* = 6.0), 6.88 – 6.92 (m, 4H), 6.86 (d, 1H, *J* = 9.0), 6.73 (d, 1H, *J* = 3.0), 6.66 – 6.72 (m, 2H), 6.64 (dd, 2H, *J* = 9.0, *J* = 3.0), 6.43 – 6.48 (m, 3H), 6.35 – 6.43 (m, 3H), 6.32 (d, 1H, *J* = 3.0), 6.26 (d, 1H, *J* = 3.0), 6.10 (s, 1H), 4.49 (s, 2H), 4.40 – 4.47 (m,

8H), 4.07 (s, 2H), 4.00 (s, 2H), 3.97 (s, 2H), 3.93 (d, 2H, *J* = 6.0), 3.87 – 3.91 (m, 4H), 3.72 – 3.81 (m, 8H), 1.58 – 1.82 (m, 5H), 1.18 – 1.55 (m, 40H), 0.79 – 0.95 (m, 30H);

^{13}C NMR (63 MHz, $CDCl_3$): δ_C = 161.7, 150.1, 149.9, 149.8, 149.7, 149.2, 149.1, 149.0, 148.9, 148.3, 142.7, 142.6, 142.5, 142.0, 141.4, 128.1, 127.3, 126.9, 126.6, 124.7, 123.2, 122.0, 121.9, 114.3, 113.8, 113.3, 113.2, 113.0, 112.5, 112.4, 112.0, 111.9, 111.7, 110.6, 99.6, 71.9, 71.6, 70.9, 70.8, 55.1, 55.0, 54.3, 54.1, 53.6, 51.4, 51.2, 50.9, 50.2, 39.7, 39.6, 39.3, 30.7, 30.6, 29.2, 29.1, 24.1, 24.0, 23.2, 23.1, 14.2, 14.1, 11.3, 11.2;

MS (ES+): m/z (%) = 1622 (20) [M + H^+], 1644 (100) [M + Na^+];

HRMS (ES+): calculated for $C_{101}H_{138}N_9O_9$ 1621.0618, found 1621.0594;

FT-IR (thin film): ν_{max}/cm^{-1} 2958, 2927, 2859, 1599, 1503, 1464, 1414, 1340, 1264, 1225, 1065, 968.

Synthesis of 9

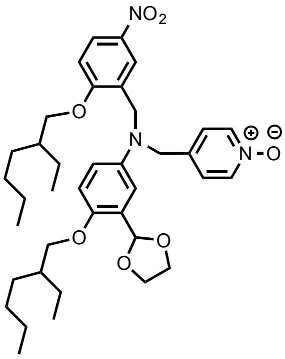

3 (0.19 g, 0.48 mmol, 1 equiv.) and **4** (0.56 g, 1.99 mmol, 4 equiv.) were dissolved in $CHCl_3$ (1.7 mL) and $NaBH(OAc)_3$ (0.28 g, 1.34 mmol, 2.8 equiv.) was added at room temperature with stirring. After 18 h the reaction was quenched with saturated aqueous $NaHCO_3$ solution, and extracted into $CHCl_3$ (4 × 10 mL). All the organic fractions were washed with water (1 × 10 mL), brine (1 × 10 mL) and dried ($MgSO_4$) before the solvent removed on a rotary evaporator. The crude product was purified using flash chromatography on silica eluting with a gradient from 0 to 4% of MeOH in DCM to yield a golden yellow oil (0.305 g, 96%).

^{1}H NMR (400 MHz, $CDCl_3$): δ_H = 8.17 (d, 2H, *J* = 6.5), 8.16 (dd, 1H, *J* = 6.0, *J* = 3.0) 8.08 (d, 1H, *J* = 3.0) 7.20 (d, 2H, *J* = 6.5) 6.95 (d, 1H, *J* = 3.0), 6.94 (d, 1H, *J* = 9.0) 6.76 (d, 1H, *J* = 9.0) 6.58 (dd, 1H, *J* = 9.0, *J* = 3.0) 6.07 (s, 1H) 4.57 (s, 2H,) 4.51 (s, 2H,) 4.03 – 3.93 (m, 6H) 3.81 (d, 2H, ^3J = 5.5) 1.83 – 1.68 (m, 2H) 1.54 – 1.24 (m, 16H) 0.98 – 0.84 (m, 12H);

^{13}C NMR (101 MHz, $CDCl_3$): δ_C = 162.1, 151.2, 141.8, 141.6, 139.4, 127.8, 127.3, 125.1, 124.8, 123.7, 115.8, 113.7, 113.1, 110.9, 99.4, 71.8, 65.4, 54.3, 51.0, 39.6, 39.4, 30.7, 29.2, 24.1, 23.3, 23.2, 14.3, 11.3;

HRMS (ES+): calculated for $C_{38}H_{54}N_3O_7$ 664.3962, found 664.3990;

FT-IR (thin film): ν_{max}/cm^{-1} 3020, 2962, 2931, 2401, 2254, 1594, 1516, 1484, 1382, 1342, 1266, 1216, 1083, 909.

Synthesis of 9'

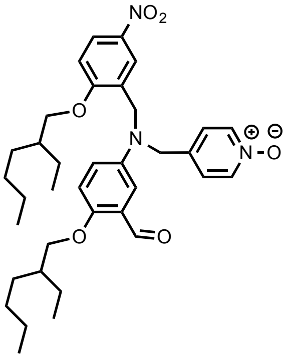

9 (0.255 g, 0.384 mmol, 1 equiv.) was dissolved in $CHCl_3$ (5 mL) and concentrated aqueous HCl (5 mL) was added with stirring. After 2 days the mixture was neutralised using aqueous $NaHCO_3$ and the organic portion separated from the aqueous part. The aqueous layer was washed with $CHCl_3$ (3 × 10 mL) before all organic fractions were washed with brine (1 × 10 mL) dried ($MgSO_4$) and the solvent removed using a rotary evaporator to yield a yellow oil (0.240 g, 99%) requiring no further purification.

^{1}H NMR (400 MHz, CDCl$_3$): δ_H = 10.41 (s, 1H), 8.17 – 8.11 (m, 3H), 7.94 (d, 1H, *J* = 3.0), 7.16 (d, 1H, *J* = 7.0), 7.11 (d, 1H, *J* = 3.0), 6.94 (d, 2H, *J* = 9.0), 6.86 (d, 1H, *J* = 9.0), 6.82 (dd, 1H, *J* = 9.0, *J* = 3.0) 4.58 (s, 2H), 4.54 (s, 2H), 3.98 (d, 2H, *J* = 5.5), 3.87 (d, 2H, *J* = 5.5), 1.83 – 1.66 (m, 2H), 1.53 – 1.18 (m, 16H), 0.96 – 0.80 (m, 12H);

^{13}C NMR (101 MHz, CDCl$_3$): δ_C = 189.8, 162.1, 155.5, 141.8, 141.5, 139.4, 137.9, 127.0 , 125.5, 125.2, 124.5, 123.1, 121.5, 114.5, 111.7, 110.9, 71.8, 71.4, 53.8, 50.6, 39.6, 39.3, 30.7, 30.6, 29.2, 29.1, 24.1, 24.1, 23.1, 23.1, 14.2, 14.2 , 11.3, 11.2;

HRMS (ES+): calculated for $C_{36}H_{50}N_3O_6$ 620.3700, found 620.3704;

FT-IR (thin film): ν_{max}/cm^{-1} 3055, 2988, 2306, 1422, 1265, 896.

Synthesis of 10

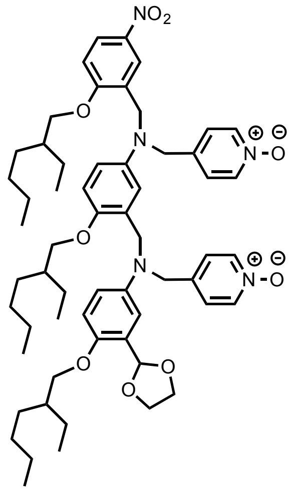

9 (0.50 g, 0.80 mmol, 1 equiv.) and **3** (0.48 g, 1.2 mmol, 1.5 equiv.) were dissolved in $CHCl_3$ (2.9 mL) with stirring and STAB-H (0.476 g, 2.3 mmol, 2.8 equiv.) was added at room temperature. After 18 h the reaction was quenched with saturated aq. $NaHCO_3$ solution, and extracted into $CHCl_3$ (4 × 10 mL). All the organic fractions were washed with water (1 × 10 mL), brine (1 × 10 mL) and dried ($MgSO_4$) before the solvent removed using a rotary evaporator. The crude product was purified using flash chromatography on silica eluting with a gradient of 0 to 10% of MeOH in a 1:1 mixture of acetonitrile and $CHCl_3$ to yield a golden yellow oil (0.47 g, 59%).

^{1}H NMR (500 MHz, $CDCl_3$): δ_H = 8.08 (d, 4H, *J* = 6. and *J* = 6.5), 8.07 (dd, 1H, *J* = 9.0, *J* = 3.0), 7.88 (d, 1H, *J* = 3.0) 7.09 (d, 2H, *J* = 6.5), 7.02 (d, 2H, *J* = 6.5), 6.88 (d, 1H, *J* = 9.0), 6.75 (d, 1H, *J* = 3.0), 6.72 (d, 1H, *J* = 9.0), 6.65 (d, 1H, *J* = 9.0), 6.46 (dd, 1H, *J* = 9.0, *J* = 3.0), 6.39 (dd, 1H, *J* = 9.0, *J* = 3.0), 6.32 (d, 1H, *J* = 3.0), 6.04 (s, 1H), 4.48 (s, 2H), 4.45 (s, 2H), 4.41 (s, 2H), 4.19 (s, 2H) 4.03 – 3.93 (m, 4H), 3.94 (d, 2H, *J* = 5.5), 3.82 (d, 2H, *J* = 5.5) 3.76 (d, 2H, *J* = 5.5), 1.80 – 1.70 (m, 2H), 1.69 – 1.61 (m, 1H), 1.18 – 1.56 (m, 24H), 0.97 – 0.80 (m, 18H);

^{13}C NMR (126 MHz, $CDCl_3$): δ_C = 161.6 , 150.1, 150.0, 142.0, 141.2, 139.1, 138.5, 127.3, 126.6, 124.7, 124.3, 122.9, 114.4, 113.5, 112.5, 112.3, 111.6, 110.7, 99.1, 71.7, 71.5,

70.7, 65.1, 54.6, 53.1, 50.8, 49.9, 39.5, 39.4, 39.2, 30.6, 30.5, 29.0, 24.0, 23.9, 23.0, 22.9, 11.1;

HRMS (ES+): calculated for $C_{59}H_{81}N_5O_9Na$ 1026.5932, found 1026.5944;

FT-IR (thin film): ν_{max}/cm^{-1} 3686, 3020, 2401, 1520, 1477, 1424, 1216, 1020, 929, 909.

4.1.1.1 Synthesis of 10’

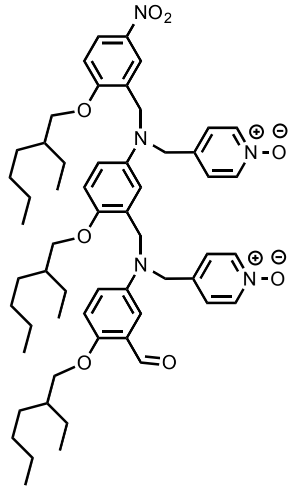

10 (0.36 g, 0.36 mmol, 1 equiv.) was dissolved in $CHCl_3$ (10 mL) and concentrated aqueous HCl (10 mL) was added with stirring. After 2 days the mixture was neutralised using aqueous $NaHCO_3$ and the organic portion separated from the aqueous part. The aqueous layer was washed with $CHCl_3$ (3 × 10 mL) before all organic fractions were washed with brine (1 × 10 mL) and dried ($MgSO_4$) to yield a bright viscous yellow oil (0.34 g, 97%) requiring no further purification.

^{1}H NMR (500 MHz, $CDCl_3$): δ_H = 10.40 (s, 1H), 8.09 – 8.06 (m, 4H), 8.04 (dd, 1H, *J* = 9.0, *J* = 3.0), 7.82 (d, 1H, *J* = 3.0), 7.07 – 7.02 (m, 4H), 6.90 (d, 1H, *J* = 3.0), 6.81 (d, 1H, *J* = 9.0), 6.72 (d, 1H, *J* = 9.0), 6.69 (d, 1H, *J* = 9.0), 6.59 (dd, 1H, *J* = 9.0, *J* = 3.0), 6.47 (dd, 1H, *J* = 9.0, *J* = 3.0), 6.26 (d, 1H, *J* = 3.0), 4.46 (s, 2H), 4.45 (s, 2H), 4.42 (s, 2H,), 4.26 (s, 2H), 3.93 – 3.84 (m, 4H), 3.76 (d, 2H, *J* = 5.5), 3.73 – 3.69 (m, 4H), 1.79 – 1.61 (m, 3H), 1.55 – 1.19 (24H, m), 0.97 – 0.82 (m, 18H);

^{13}C NMR (126 MHz, $CDCl_3$): δ_C = 189.7, 161.5, 154.6, 150.0, 141.7, 141.2, 141.2, 139.2, 139.1, 138.1, 127.2, 126.1, 125.0, 124.6, 124.2, 124.1, 122.8, 120.5, 114.0, 113.2, 112.5, 112.5, 110.6, 110.0, 71.6, 71.4, 70.7, 54.4, 53.0, 50.8, 50.0, 39.5, 39.4, 39.1, 30.6, 30.6, 30.5, 29.1, 29.0, 29.0, 24.0, 23.9, 23.9, 23.0, 23.0, 22.9, 14.0, 14.0, 14.0, 11.2, 11.1, 11.1;

HRMS (ES+): calculated for $C_{57}H_{78}N_5O_8$ 960.5850, found 960.5817;

FT-IR (thin film): ν_{max}/cm^{-1} 3020, 2400, 1521, 1425, 1265, 1217, 909.

Synthesis of 11

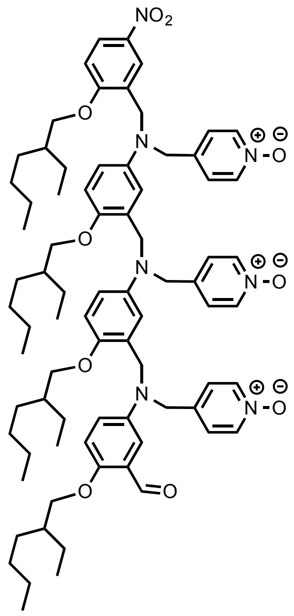

10 (0.36 g, 0.37 mmol, 1 equiv.) and **3** (0.26 g, 0.65 mmol, 2.0 equiv.) were dissolved in $CHCl_3$ (2 mL) and $NaBH(OAc)_3$ (0.19 g, 0.91 mmol, 2.8 equiv.) was added with stirring. After 5 days the reaction was quenched with saturated aqueous $NaHCO_3$ solution, and extracted into $CHCl_3$ (4 × 10 mL). All the organic fractions were washed with water (1 × 10 mL), brine (1 × 10 mL) and dried ($MgSO_4$) before the solvent removed with a rotary evaporator. The crude product was purified using flash chromatography on silica eluting with a gradient from 0% to 10% MeOH in a 2:8 mixture of acetonitrile and $CHCl_3$ to yield a mixture of the expected product and the benzaldehyde derivative. This mixture was dissolved in $CHCl_3$ (5 mL) and concentrated aqueous HCl (5 mL) was added with stirring. After 2 days the mixture was neutralised using aqueous $NaHCO_3$ and the organic portion separated from the aqueous part. The aqueous layer was washed with $CHCl_3$ (3 × 10 mL) before all organic fractions were washed with brine (1 × 10 mL) dried ($MgSO_4$) and the solvent removed using a rotary evaporator. The crude mixture was then purified via flash chromatography on silica eluting with a gradient from 5% to 50% of MeOH in DCM to yield a viscous red oil (0.073 g, 15%).

^{1}H NMR (400 MHz, CDCl$_3$): δ_H = 10.43 (s, 1H), 8.12 (dd, 1H, *J* = 9.0, *J* = 3.0), 8.09 (d, 2H, *J* = 7.0), 8.04 – 7.99 (m, 4H), 7.91 (d, 1H, *J* = 3.0), 7.10 – 7.04 (m, 3H), 7.00 (d, 2H, *J* = 7.0), 6.97 (d, 2H, *J* = 7.0), 6.93 (d, 1H, *J* = 9.0), 6.81 (d, 1H, *J* = 9.0), 6.73 – 6.66 (m, 3H), 6.47 (dd, 1H, *J* = 9.0, *J* = 3.0), 6.39 – 6.34 (m, 2H), 6.24 (d, 1H, *J* = 3.0), 4.52 – 4.47 (m, 4H), 4.43 (s, 2H), 4.41 (s, 2H), 4.24 (s, 2H), 4.16 (s, 2H), 3.99 (d, 2H, *J* = 5.5), 3.91 (d, 2H, *J* = 5.5), 3.85 (d, 2H, *J* = 5.5), 3.74 (d, 2H, *J* = 5.5), 1.82 – 1.60 (m, 4H), 1.55 – 1.22 (m, 32H), 0.98 – 0.83 (m, 24H);

^{13}C NMR (126 MHz, CDCl$_3$): δ_C = 189.7, 161.7, 154.8, 149.9, 149.5, 142.1, 141.7, 141.3, 141.2, 139.2, 139.0, 138.9, 138.6, 138.4, 127.6, 126.8, 126.7, 125.9, 125.1, 124.7, 124.2, 124.2, 124.1, 123.0, 121.8, 120.8, 114.1, 113.5, 112.9, 112.5, 112.4, 112.3, 110.9, 110.6, 71.6, 71.4, 70.9, 70.8, 54.3, 53.5, 53.1, 50.9, 50.8, 50.6, 39.5, 39.4, 39.2, 30.6, 30.5, 29.7, 29.1, 29.0, 24.0, 23.9, 23.0, 14.1, 11.2;

HRMS (ES+): calculated for $C_{78}H_{105}O_{10}N_7Na$ 1322.7815, found 1322.7782;

FT-IR (thin film): ν_{max}/cm^{-1} 2959, 2925, 2872, 1682, 1502, 1483, 1340, 1264, 1245, 1229.

Synthesis of 12

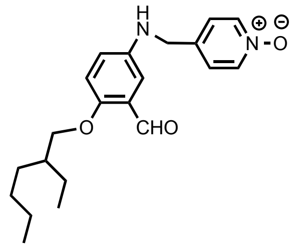

3 (0.5 g, 1.2 mmol) was dissolved in $CHCl_3$ (10 mL) and concentrated aqueous HCl (10 mL) was added with stirring. After 2 days the mixture was neutralised using aqueous $NaHCO_3$ and the organic portion separated from the aqueous part. The aqueous layer was washed with $CHCl_3$ (3 × 10 mL) before all organic fractions were washed with brine (1 × 10 mL) dried ($MgSO_4$) and the solvent removed using a rotary evaporator. The crude mixture was then purified via flash chromatography on silica eluting with a gradient from 2 to 5% of MeOH in MeCN/$CHCl_3$ 50% to yield a viscous red oil (0.3 g, 66 %).

^{1}H NMR (500 MHz; $CDCl_3$): δ_H 10.46 (s, 1H), 8.17 (d, *J* = 7.0, 2H), 7.29 (d, *J* = 7, 2H), 7.05 (d, *J* = 3.0, 1H), 6.89 (d, *J* = 9, 1H), 6.82 (dd, *J* = 9, *J* = 3.0, 1H), 4.36 (s, 2H), 4.18 (s, broad, 1H), 3.91-3.90 (m, 2H), 1.78-1.73 (m, 1H), 1.54-1.31 (m, 8H), 0.96-0.90 (m, 6H).

^{13}C NMR (126 MHz; CDCl3): δ_C 189.8, 155.5, 140.7, 139.2, 138.9, 125.4, 124.5, 121.4, 114.4, 110.6, 71.4, 46.8, 39.5, 30.6, 29.1, 23.9, 23.0, 14.1, 11.2.

MS (ES+): m/z (%) = 357.2 (100) [M + H^+],

HRMS (ES+): calculated for $C_{21}H_{29}N_2O_3$ 357.2178, found 357.2182;

FT-IR (ATR): ν_{max}/cm^{-1} 3267, 2956, 2928, 2863, 1700, 1494, 1395, 1233, 1180.

M.p. 153-155 °C

Synthesis of 13

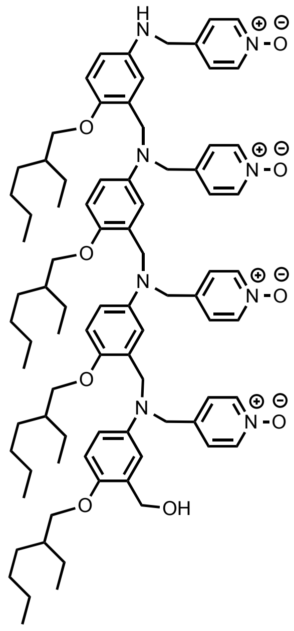

12 (260 mg, 0.7 mmol, 1 equiv.) was dissolved in DCE (0.5 mL) and $NaBH(OAc)_3$ (410 mg, 2.8 equiv.) was added with stirring. After 48 h the solution was filtered and the solvent removed by rotary evaporator. This crude mixture was dissolved in MeOH (1 mL) and then $NaBH_4$ (0.2 g, 7 mmol, 10 equiv.) was added slowly at 0 °C with stirring. This mixture was stirred for 1 hour before the solution was neutralized using concentrated aqueous HCl. This solution was washed with EtOAc (5 × 10 mL) and all the organic extracts were combined and washed with brine (1 × 10 mL) and then dried ($MgSO_4$). The crude oil was purified via flash chromatography on C_{18} eluting with a gradient from 70% to 100% MeOH in MeCN to yield a golden oil (17 mg , 2%).

^{1}H NMR (500 MHz; $CDCl_3$): δ_H 8.06 (d, *J* =7.0, 2H), 8.01 (d, *J* = 7.1, 2H), 7.94 (d, *J* = 7.0, 2H), 7.88 (d, *J* = 7.0, 2H), 7.11 (d, *J* = 7.1, 2H), 7.06 (d, *J* = 7.1, 2H), 6.93 (d, *J* = 7.1, 2H), 6.86 (d, *J* = 7.0, 2H), 6.73-6.63 (m, 6H), 6.52-6.47 (m, 2H), 6.40 (dd, *J* = 9.0, *J* = 3.0, 1H), 6.30-6.25 (m, 4H), 4.61 (s, 2H), 4.45-4,43 (m, 6H), 4.21 (s, 2H), 4.16 (s, 2H), 4.14 (s,

2H), 4.03 (s, 2H), 3.80-3.74 (m, 9H), 1.70-1.63 (m, 4H), 1.49-1.25 (m, 32H), 0.92-0.84 (m, 24H).

^{13}C NMR (126 MHz; CDCl3): δ_C 149.8, 149.40, 149.35, 149.1, 142.3, 142.06, 142.04, 140.8, 139.82, 139.79, 139.17, 139.03, 139.00, 138.97, 138.87, 138.85, 131.7, 127.01, 126.99, 126.7, 124.47, 124.41, 124.23, 123.9, 113.19, 113.08, 112.94, 112.63, 112.55, 112.47, 112.28, 112.27, 112.08, 111.81, 111.73, 71.05, 70.93, 70.88, 70.85, 60.6, 53.87, 53.74, 53.62, 50.87, 50.75, 49.8, 47.1, 39.55, 39.50, 39.46, 30.6, 29.109, 29.10, 29.06, 24.0, 23.0, 14.10, 14.08, 11.19, 11.17

MS (ES+): m/z (%) = 1380 (40) [M + H^+], 1402 (100) [M + Na],;

HRMS (ES+): calculated for $C_{84}H_{115}N_8O_9$ 1379.8782, found 1379.8757;

FT-IR (ATR): ν_{max}/cm^{-1} 3346, 2957, 2926, 2859, 1502, 1356, 1225, 1170, 1034.

Synthesis of 14

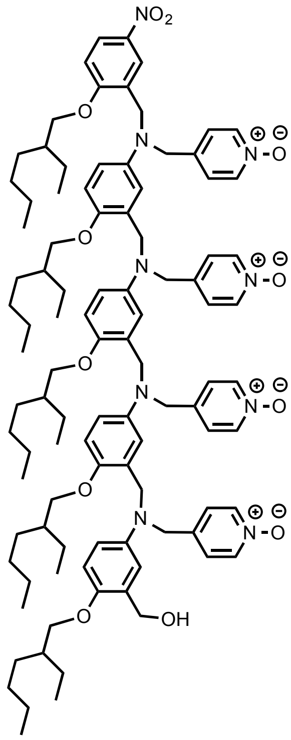

13 (17 mg, 0.01 mmol, 1 equiv.) and **4** (5.6 mg, 0.02 mmol, 2 equiv.) were dissolved in DCE (0.1 mL) and $NaBH(OAc)_3$ (8.0 mg, 0.04 mmol, 3.2 equiv.) was added with stirring. After 18 hours the reaction was quenched with saturated aqueous $NaHCO_3$ solution, and extracted into $CHCl_3$ (4 × 10 mL). All the organic fractions were washed with water (1 × 10 mL), brine (1 × 10 mL) and dried ($MgSO_4$) before the solvent removed with a rotary evaporator. The crude product was purified using flash chromatography on silica eluting with a gradient from 0% to 10% of MeOH in EtOAc to yield a golden yellow oil (5.0 mg, 30%).

^{1}H NMR (500 MHz; $CDCl_3$): δ_H 8.12-8.09 (m, 3H), 7.94-7.84 (m, 8H), 7.09 (d, *J* = 7.0, 2H), 6.93 (d, *J* = 7.0, 2H), 6.91, 6.86 (m, 4H), 6.71-6.68 (m, 4H), 6.63 (d, *J* = 9, 1H), 6.48-6.43 (m, 3H), 6.33 (d, *J* = 3, 1H), 6.23 (dd, *J* = 9, *J* =3, 1H), 6.16 (dd, *J* = 9, *J* = 3, 2H),

4.62 (s, 2H), 4.49 (s, 2H), 4.46-4.42 (m, 6H), 4,37 (s, 2H), 4.19 (s, 2H), 4.00-3.96 (m, 6H), 3.79-3.72 (m, 8H), 1.78-1.73 (5H, m,) 1.50-1.24 (m, 40H), 0.93-0.83 (m, 30H).

^{13}C NMR (126 MHz; CDCl3): δ_C 161.7, 150.0, 149.4, 149.3, 149.0, 141.9, 141.9, 141.8, 141.2, 141.1, 139.6, 139.2, 139.1, 138.9, 138.7, 138.5, 131.9, 127.5, 127.0, 126.8, 126.6, 125.8, 124.8, 124.4, 124.3, 124.1, 123.8, 123.0, 113.7, 112.8, 112.6, 112.5, 112.3, 112.3, 111.8, 111.3, 110.7, 71.6, 71.1, 70.9, 70.8, 70.7, 60.3, 54.5, 53.6, 53.4, 53.2, 51.1, 50.9, 49.5, 39.5, 39.5, 39.4, 39.2, 30.6, 30.6, 30.5, 29.1, 29.1, 29.0, 24.0, 23.9, 23.0, 23.0, 22.9, 14.1, 14.1, 11.2, 11.1

MS (ES+): m/z (%) = 822 (70) [M +2H], 1643 (30)[M +H], 1665 (100) [M +Na],

HRMS (ES+): calculated for $C_{99}H_{136}N_9O_{12}$ 1643.0303, found 1643.0314;

FT-IR (ATR): ν_{max}/cm^{-1} 2957, 2922, 2853, 1612, 1591, 1503, 1483, 1464, 1340, 1227, 1168, 1031.

NMR Binding studies

All association constants were measured by ^{1}H NMR titrations. The host was dissolved in toluene-d8 at a known concentration. The guest was dissolved in the host stock solution at a known concentration. A known volume of host was added to an NMR tube and the spectrum was recorded. Known volumes of guest were added to the tube, and the spectrum was recorded after each addition. The chemical shifts of the host were monitored as a function of guest concentration and analysed using purpose written software in Microsoft Excel®. Errors were calculated as two times the standard deviation from the average value from repetitions.

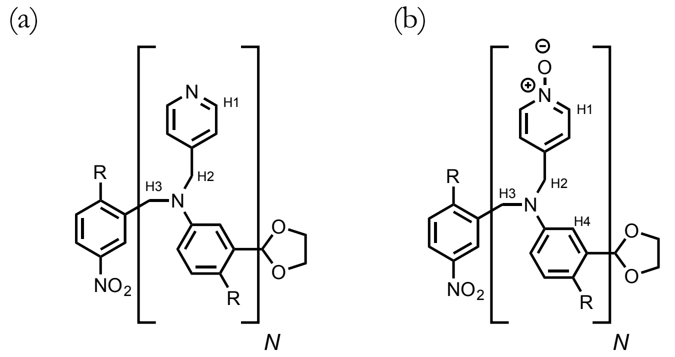

Figure S1 Proton labelling scheme for (a) pyridine oligomers and (b) pyridine N-oxide oligomers. R = 2-ethylhexoxy.

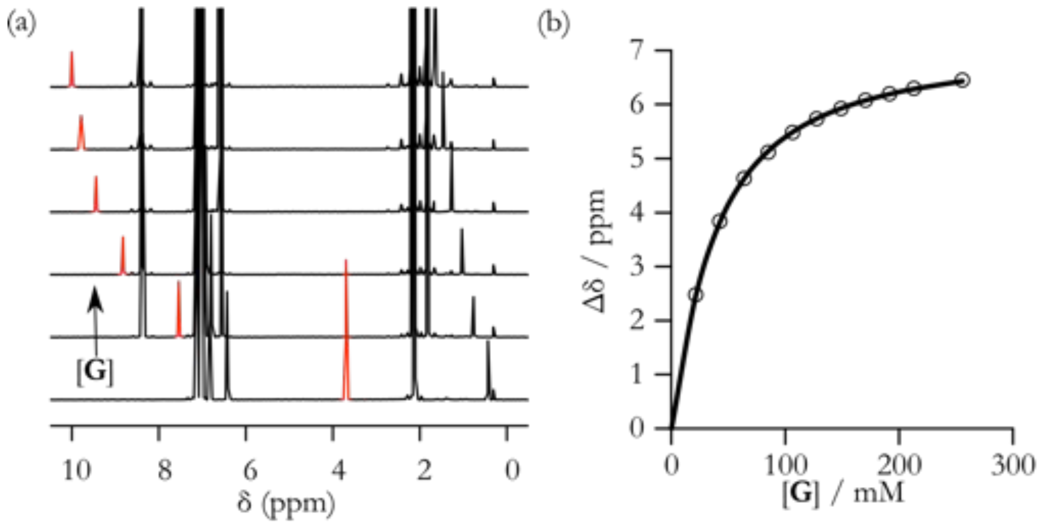

Figure S2. Titration of 4-methylpyridine into 4-methylphenol (19 mM) at 298 K in toluene-d8. (a) 400 MHz ^{1}H NMR spectra (the OH signal is highlighted in red). (b) Chemical shift change as a function of guest concentration (the line represents the best fit to a 1:1 binding isotherm G= 4-methylpyridine).

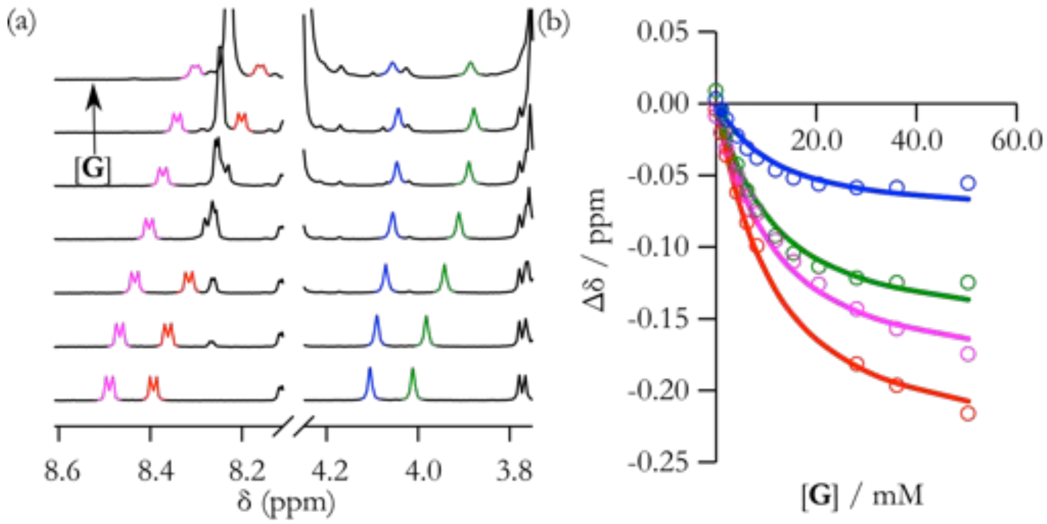

Figure S3 Titration of DD into **6** (5.0 mM) at 298 K in toluene-d8. (a) 400 MHz ^{1}H NMR spectra. (b) Chemical shift change as a function of guest concentration (the line represents the best fit to a 1:1 binding isotherm). The magenta and red signals correspond to H1; the blue and green signals correspond to H2 (see Figure S1(a) for labelling scheme).

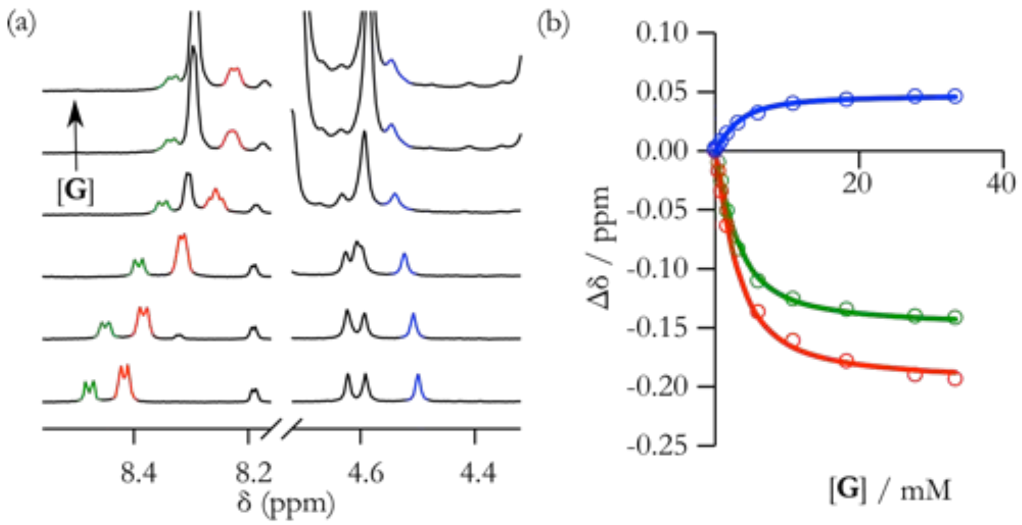

Figure S4 Titration of DDD into **7** (3.0 mM) at 298 K in toluene-d8. (a) 400 MHz ^{1}H NMR spectra. (b) Chemical shift change as a function of guest concentration (the line represents the best fit to a 1:1 binding isotherm). The green and red signals correspond to H1 and blue corresponds to H3 (see Figure S1(a) for labelling scheme).

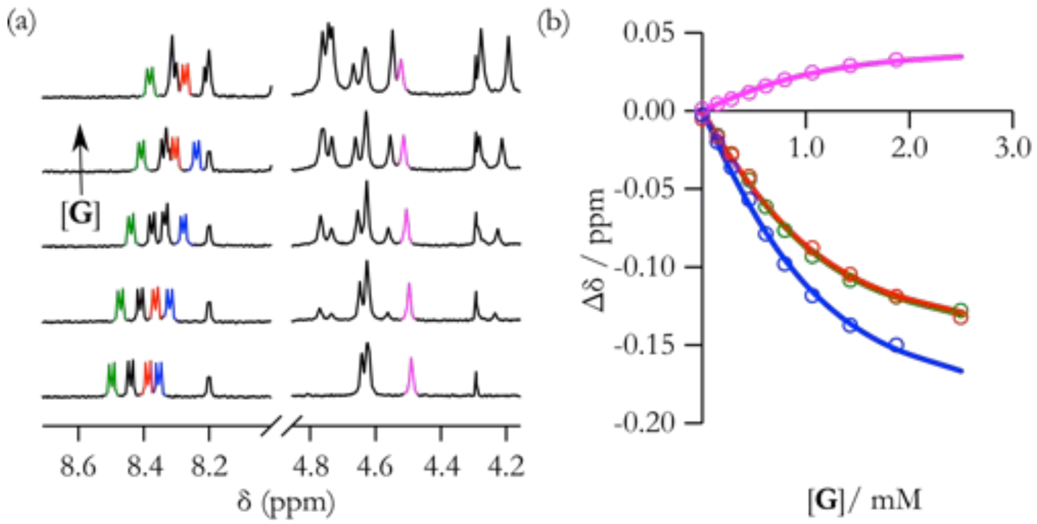

Figure S5 Titration of DDDD into **8** (1.0 mM) at 298 K in toluene-d8. (a) 400 MHz ^{1}H NMR spectra. (b) Chemical shift change as a function of guest concentration (the line represents the best fit to a 1:1 binding isotherm). The green red and blue signals correspond to H1, magenta corresponds to H3 (see Figure S1(a) for labelling scheme).

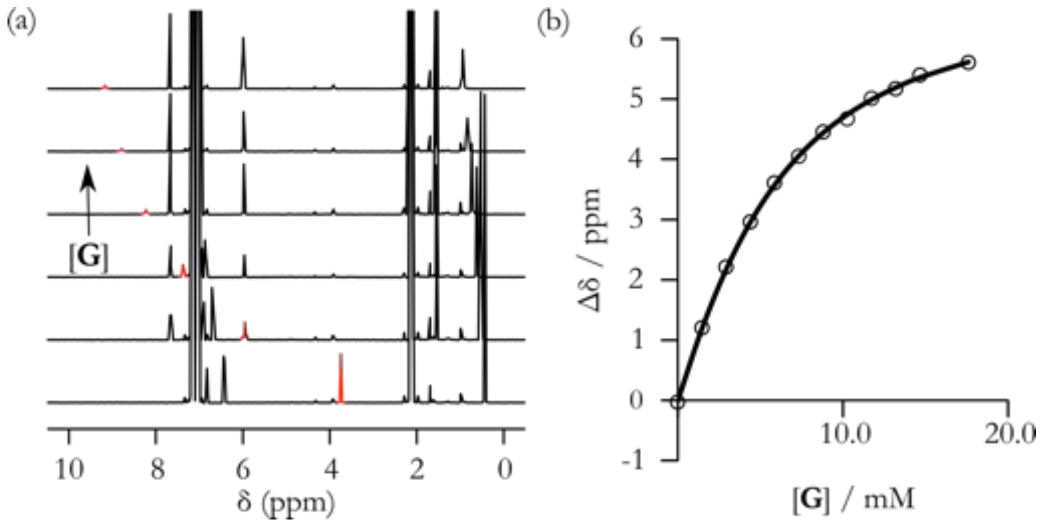

Figure S6 Titration of methylpyridine *N*-oxide into 4-methylphenol (4.5 mM) at 298 K in toluene-d8. (a) Example 400 MHz ^{1}H NMR spectra (OH signal highlighted in red). (b) Plot of the change in chemical shift of the OH signal as a function of guest concentration (the line represents the best fit to a 1:1 binding isotherm).

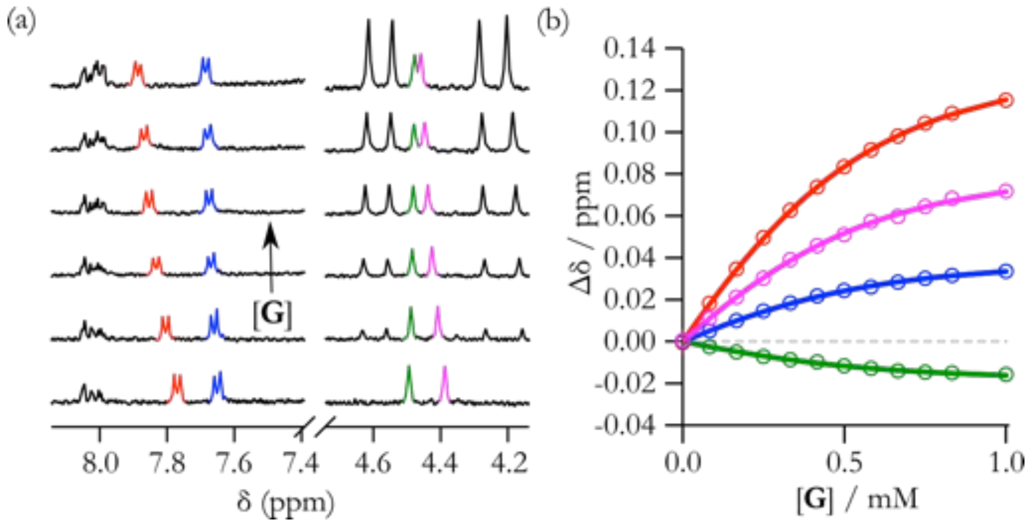

Figure S7 Titration of DD into **10** (0.5 mM) at 298 K in toluene-d8. (a) Example 400 MHz ^{1}H NMR spectra. (b) Plot of the change in chemical shift as a function of guest concentration (the line represents the best fit to a 1:1 binding isotherm). The red and blue signals correspond to H1 and magenta and green signals correspond to H3 (see Figure S1(b) for labelling scheme).

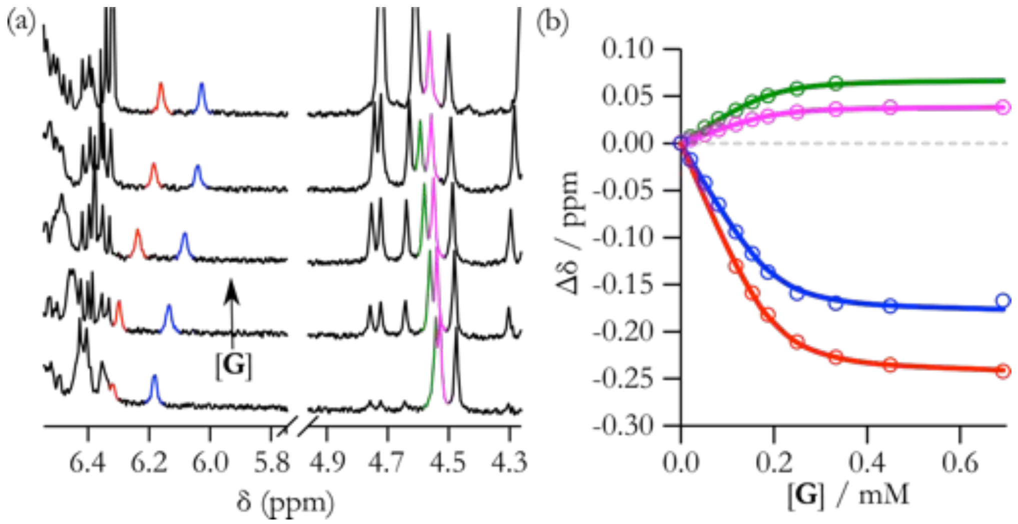

Figure S8 Titration of DDD into **11** (0.2 mM) at 298 K in toluene-d8. (a) Example 400 MHz ^{1}H NMR spectra. (b) Plot of the change in chemical shift as a function of guest concentration (the line represents the best fit to a 1:1 binding isotherm). The red and blue signals correspond to H4 and magenta and green signals correspond to H3 (see Figure S1(b) for labelling scheme).

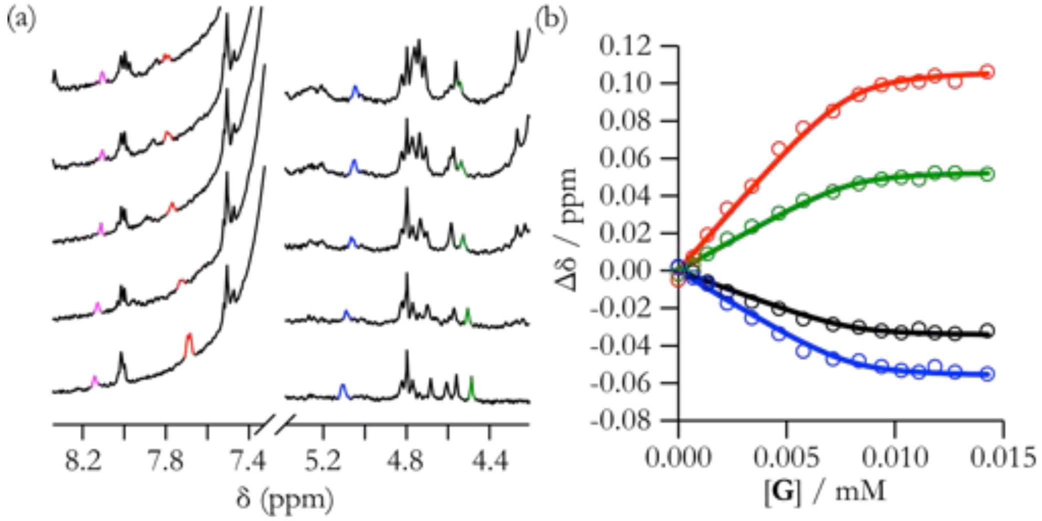

Figure S9 Titration of DDDD into **14** (0.008 mM) at 298 K in toluene-d8. (a) Example 500 MHz ^{1}H NMR spectra. (b) Plot of the change in chemical shift as a function of guest concentration (the line represents the best fit to a 1:1 binding isotherm). The red signals correspond to H1 and green signals correspond to H3 (see Figure S1(b) for labelling scheme).

Table S1 ^{1}H NMR chemical shifts of the free pyridine host (ppm) obtained by fitting the pyridine (A)-phenol (D) titration data to a 1:1 binding isotherm.[a]

proton	AA•DD	AAA•DDD	AAAA•DDD
H1	8.51	8.48	8.47
	8.40	8.43	8.41
		8.43	8.34
			8.33
H2	4.10	4.15	4.06
	4.00	3.95	3.92
		3.90	3.93
			3.89
H3	4.58	4.62	4.63
	4.45	4.59	4.59
		4.50	4.59
			4.49

a refer to Figure S1(a) for proton labelling scheme

Table S2 Limiting ^{1}H NMR chemical shifts of the fully bound pyridine host (ppm) obtained by fitting the pyridine (A)-phenol (D) titration data to a 1:1 binding isotherm.[a]

proton	AA•DD	AAA•DDD	AAAA•DDDD
H1	8.31	8.32	8.33
	8.16	8.23	8.25
		8.23	8.21
			8.15
H2	4.02	4.14	4.04
	3.84	3.73	3.81
		3.63	3.79
			3.66
H3	4.55	4.63	4.63
	4.47	4.60	4.59
		4.55	4.59
			4.49

a refer to Figure S1(a) for numbering scheme.

Table S3 Limiting complexation-induced changes in pyridine (A) host ^{1}H NMR chemical shift (ppm) obtained by fitting pyridine-phenol titration data to a 1:1 binding isotherm.[a]

proton	Complex		
	AA•DD	AAA •DDD	AAAA•DDDD
H1	-0.19	-0.16	-0.14
	-0.24	-0.20	-0.16
		-0.20	-0.14
			-0.18
H2	-0.08	-0.01	-0.02
	-0.16	-0.22	-0.11
		-0.27	-0.13
			-0.23
H3	-0.02	0.01	0.03
	0.02	0.01	0.01
		0.05	0.01
			0.04

a refer to Figure S1(a) for numbering scheme;

Table S4 ^{1}H NMR chemical shifts of the free pyridine *N*-oxide host host (ppm) obtained by fitting the pyridine *N*-oxide (A)-phenol (D) titration data to a 1:1 binding isotherm.[a]

proton	**AA•DD**	**AAA•DDD**	**AAAA•DDDD**
	7.78	7.75	b
H1	7.66	7.68	7.69
		7.64	b
	b	3.96	b
H2	b	3.87	b
		3.75	b
	4.49	4.54	4.48
H3	4.39	4.52	b
		4.47	b
H4	6.33	6.40	b
		6.20	b

a refer to Figure S1(b) for numbering scheme; b signal could not be followed due to signal overlap.

Table S5 Limiting ^{1}H NMR chemical shifts of the fully bound pyridine *N*-oxide host (ppm) obtained by fitting the pyridine (A)-phenol (D) titration data to a 1:1 binding isotherm.[a]

proton	**AA•DD**	**AAA•DDD**	**AAAA•DDDD**
H1	7.93	7.81	b
	7.70	7.84	7.80
		7.57	b
H2	b	3.92	b
	b	3.84	b
		3.55	b
H3	4.47	4.61	4.54
	4.48	4.56	b
		4.50	b
H4	6.09	6.14	b
		6.02	b

a refer to Figure S1(b) for numbering scheme; b signal could not be followed due to signal overlap.

Table S6 Limiting complexation-induced changes in pyridine N-oxide ^{1}H NMR chemical shift (ppm) obtained by fitting the pyridine (A)-phenol (D) titration data to a 1:1 binding isotherm.[a]

proton	Complex		
	AA•DD	AAAA•DDD	AAAA•DDDD
H1	0.15	0.05	b
	0.04	0.16	0.11
		-0.07	b
H2	b	-0.03	b
	b	-0.03	b
		-0.21	b
H3	-0.02	0.07	0.05
	0.10	0.04	b
		0.03	b
H4	-0.24	-0.26	b
		-0.18	b

a refer to Figure 4b for numbering scheme; b signal could not be followed due to signal overlap.
